# Accompanying Structural Transformations in Polarity Switching of Heavily Doped Conjugated Polymers

**DOI:** 10.1002/adma.202505945

**Published:** 2025-07-09

**Authors:** Eunsol Ok, Sein Chung, Seung Hyun Kim, Kitae Kim, Soohyung Park, Hoimin Kim, Yeonjin Yi, Jong Dae Jang, Hansol Lee, Hyun Ho Choi, Boseok Kang, Kilwon Cho

**Affiliations:** ^1^ Department of Chemical Engineering Pohang University of Science and Technology Pohang 37673 Republic of Korea; ^2^ Department of Physics Yonsei University 50 Yonsei‐ro, Seodaemun‐gu Seoul 03722 Republic of Korea; ^3^ Division of Nano & Information Technology KIST School University of Science and Technology (UST) Seoul 02792 Republic of Korea; ^4^ SKKU Advanced Institute of Nanotechnology (SAINT) and Department of Nano Science and Engineering Sungkyunkwan University (SKKU) Suwon 16419 Republic of Korea; ^5^ Neutron Science Division Korea Atomic Energy Research Institute 1045 Daedeok‐daero, Yuseong‐gu Daejeon 34057 Republic of Korea; ^6^ School of Chemical Biological and Battery Engineering Gachon University Seongnam 13120 Republic of Korea; ^7^ Department of Materials Engineering and Convergence Technology Gyeongsang National University Jinju 52828 Republic of Korea; ^8^ Department of Nano Engineering and Department of Semiconductor Convergence Engineering Sungkyunkwan University (SKKU) Suwon 16419 Republic of Korea

**Keywords:** charge transport, conjugated polymer, homojunction diode, lewis acid doping, polarity switching

## Abstract

Despite significant recent advancements in highly functional organic semiconductors (OSCs), the *n*‐type OSCs reported to date lag behind their *p*‐type counterparts in terms of long‐term environmental stability. As an alternative approach to *n*‐type materials, a few *p*‐type polymers have been shown to undergo dramatic transitions in their charge carrier polarity to *n*‐type through transition metal‐incorporated Lewis acid doping. Although the concept of polarity switching is promising, its unclear chemical origin—particularly from a materials science perspective—limits its potential as an *n*‐type counterpart. In this work, the chemical and structural mechanisms underlying the *p*‐to‐*n* polarity switching in a heavily doped conjugated polymer are elucidated. Using gold(III) chloride‐doped indacenodithiophene‐co‐benzothiadiazole (IDTBT) as a model system, doping‐induced thin‐film structural changes are investigated. Quantitative X‐ray photoelectron spectroscopy analysis of doped IDTBT films provides direct evidence of oxidation state changes in Au and Cl ions and confirms the covalent chlorination of the polymer backbone, establishing a direct correlation between the chemical doping mechanism and polarity switching. Finally, leveraging this polarity switching behavior, a *p‐n* homojunction organic diode is demonstrated with a rectification ratio of 10^4^–10^5^, highlighting the versatility and potential of this excessively *p*‐doped *n*‐type OSC system for tailoring charge transport properties.

## Graphical abstract

The polarity switching of heavily doped *p‐*type conjugated polymers (CPs) toward environmentally stable electron‐rich thin films is demonstrated. Multiscale analyses, incorporating spectroscopic, electrochemical, and scattering techniques, reveal structural and chemical transformations induced by excessive doping with AuCl_3_. Leveraging this polarity switching behavior, a flexible homojunction *p‐n* diode is fabricated using a single CP‐dopant system with a record‐high rectification ratio of ≈10^5^.

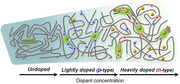

## 1 Introduction

Conjugated polymers (CPs) have garnered significant attention for their versatility and potential applications in flexible and environmentally friendly electronics.^[^ ^1^, ^2^ ^]^ However, their intrinsically low charge transport and carrier density compared to inorganic semiconductors present a major limitation. To address this issue, molecular doping has emerged as a powerful approach that enables systematic control over the electrical and optical properties of semiconducting CPs.^[^ ^3^, ^4^, ^5^ ^]^ Through molecular doping, researchers have improved thermoelectric performance, reduced contact resistance,^[^ ^6^ ^]^ and enhanced transistor stability at low doping levels,^[^ ^6^ ^]^ particularly in *p*‐type materials.^[^ ^7^, ^8^, ^9^ ^]^ Despite these advances, *n*‐type CPs—which are essential for balanced *p‐n* junction devices^[^ ^9^, ^10^ ^]^—still suffer from inadequate long‐term environmental stability. This lack of stability is primarily due to their low electron affinity, which makes them highly vulnerable to deterioration under ambient conditions.^[^ ^11^ ^]^ This issue has hindered the development of stable, high‐performance electronic devices based on CPs.

Recent efforts have proposed using *p*‐type CPs, which are generally more stable under ambient conditions, as precursors to *n*‐type materials; the previous relevant studies are listed in Table S1 (Supporting Information).^[^ ^8^, ^12^, ^13^, ^14^, ^15^, ^16^, ^17^ ^]^ This strategy leverages the inherent stability of *p*‐type materials while achieving *n*‐type characteristics via high‐concentration doping, thereby overcoming the limitations of *n*‐type CPs. The polarity switching mechanism observed in such systems has been mostly attributed to electronic band filling, where excessive doping induces the Fermi level (*E*_F_) to cross the transport level (*E*_T_), altering the Seebeck coefficient (*S*) and effectively switching the carrier type.^[^ ^8^, ^13^, ^14^, ^15^, ^16^, ^17^, ^18^ ^]^ However, while the physical perspectives are somehow plausible, the chemical dynamics of polarity switching—particularly those related to strong oxidizing agents from the materials science perspective—remain scarcely understood, especially in terms of the resulting structural and morphological changes in a CP matrix. As summarized in Table S1 (Supporting Information), previous studies have demonstrated polarity switching in CPs primarily through electronic mechanisms such as Fermi level shifts or transport gap closure induced by heavy doping. However, most of these works provide limited insight into the accompanying chemical or structural transformations. In contrast, our study highlights a chemically driven mechanism where covalent chlorination of the polymer backbone by AuCl_3_ leads to both polarity switching and structural reorganization compared to other studies as shown in Table S2 (Supporting Information). This dual role of the dopant—modifying both electronic levels and polymer morphology—offers a new perspective beyond conventional electronic‐only interpretations.

Here, we propose a mechanism for polarity switching that encompasses both the chemical and structural transformations that occur during the delocalization of charge transport within CP films under the influence of doping. In particular, we focus on the *n‐*polarity switching phenomenon during heavy *p‐*doping, with a view to gleaning valuable insights into the interplay between thin film and chemical structures of doped CPs and their charge transport behaviors. Based on the proposed mechanism, we develop a homojunction flexible *p‐n* diode with stable single‐CP *p‐n* junctions to demonstrate the usefulness of the proposed strategy. This work addresses persistent challenges underlying CP doping mechanisms, thereby contributing to the design of improved materials and processes for novel complementary devices with a soft organic homojunction.

## 2 Results and Discussion

### 2.1 Characterization of Doped IDTBT Thin Films

**Figure 1:**
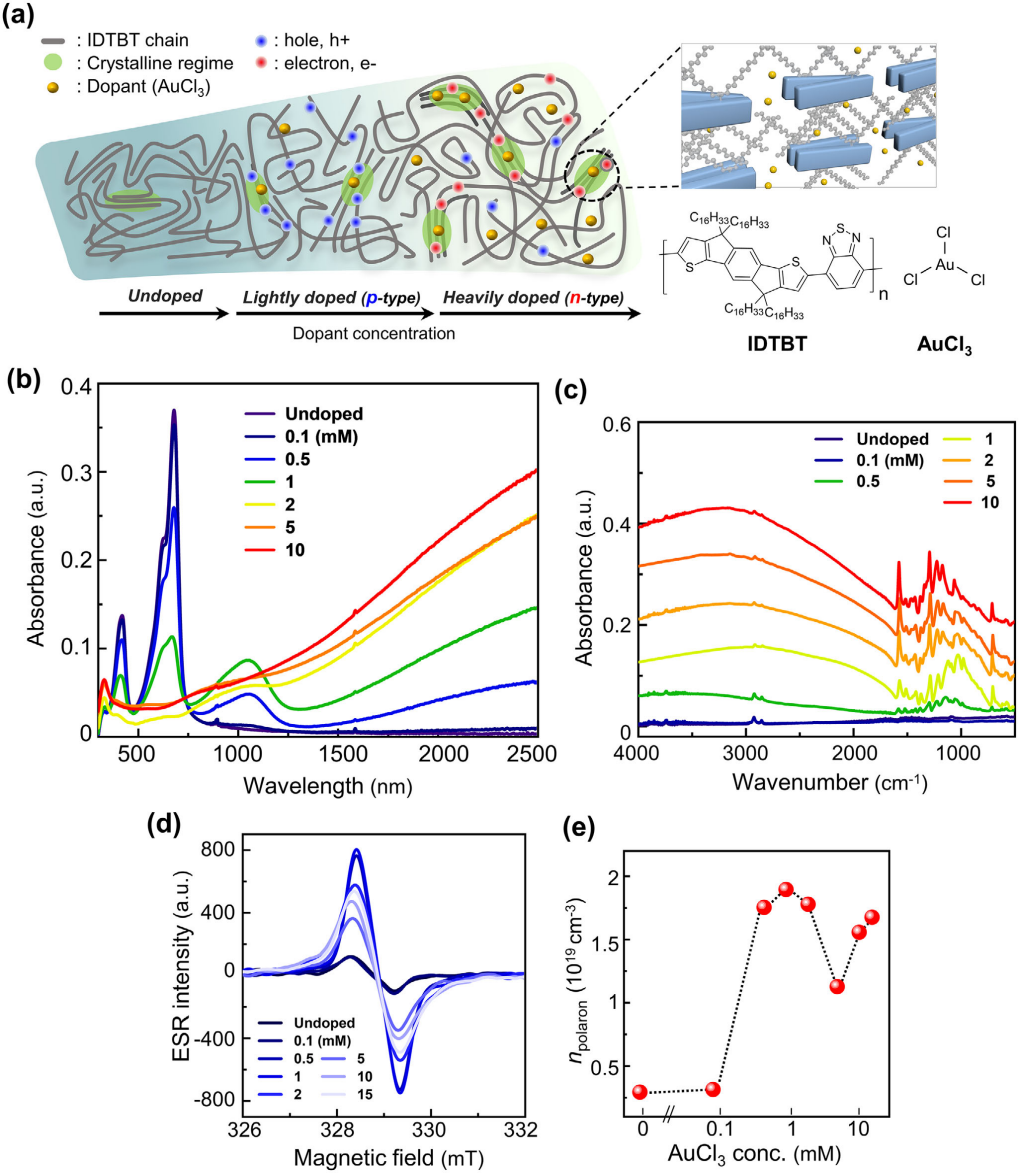
a) Schematic drawing of sequential polarity changes in undoped, lightly doped, and heavily doped IDTBT (host CP) with AuCl_3_ (dopant). b) UV–vis–NIR absorption spectra, c) FT‐IR spectra, (d) ESR spectra of doped IDTBT thin films with different doping levels. The intensity was corrected by the thickness of each sample, and the dopant concentrations are marked respectively in each figure. e) The density of polarons (*n*_polaron_) in doped IDTBT films was determined by calibrating the film dimensions using CuSO_4_·5H_2_O as a reference.

Assessing the effects of doping begins with determining the type and concentration of charge carriers in CP thin films. Sequential doping was performed using IDTBT as a host *p‐*type CP and gold(III) chloride (AuCl_3_) as a strong *p‐*type dopant.^[^ ^19^ ^]^ IDTBT, unlike highly crystalline CPs with high carrier mobility, is a near‐amorphous polymer. Instead of the frequently used FeCl_3_, AuCl_3_ was chosen for its superior thermal and environmental stabilities upon doping.^[^ ^20^ ^]^ The high thermal and environmental stabilities of AuCl_3_ can be mostly attributed to the higher redox potential and stronger oxidizing nature of the Au(III) cation compared to the Fe(III) cation, leading to stable doped states and achieving great potential for device applications. **Figure** **1**a illustrates the overall system considered in this study. It depicts the introduction of the dopant into the CP and the subsequent polarity switching, which alters both the charge carrier type and the crystallinity of the CP films, as will be discussed later. To observe these changes, the dopant concentration, denoted as [AuCl_3_], was controlled from 0.1 to 10 mM (Table S3, Supporting Information). The undoped pristine IDTBT thin film exhibited a dark sky‐blue color, but as the dopant concentration increased, the color gradually became lighter and the film became more transparent (Figure S1a, Supporting Information). In addition, the thin film thickness increased along with the doping concentration (Figure S1b, Supporting Information).

UV–vis–NIR absorption spectra provide insights into the extent of the charge‐carrier generation resulting from doping (Figure 1b). In the undoped IDTBT film, peaks corresponding to neutral states were observed at ≈400 and 600–700 nm, indicating localized π–π* transition and intramolecular charge transfer.^[^ ^21^, ^22^ ^]^ As the dopant concentration increased, the intensities of these initial neutral peaks, normalized to the film thickness, decreased gradually (Figure S2, Supporting Information). Subsequently, new absorption peaks were detected at ≈1000 nm and over 1300 nm, corresponding to [AuCl_3_] ≥ 1 mM and ≥ 5 mM, respectively. The emergence of these peaks, whose intensities increased with increasing dopant concentration, indicates the formation of polaron and bipolaron states as a result of charge transfer between the dopant and CP.^[^ ^23^, ^24^ ^]^ A similar doping trend was observed in solution‐mixing doped IDTBT solution. To further verify the stoichiometry‐dependent behavior, we examined a mixed dual‐solution system, blending AuCl_3_ and IDTBT solutions under controlled conditions (Figure S3, Supporting Information). Comparing the resulting UV–vis–NIR spectra with those from sequential doping, the spectral overlap reveals that sequential doping at 0.5–1 mM is equivalent to 5–20 wt % dopant in the dual‐solution doping protocol, whereas 2–10 mM corresponds to 25–40 wt %. These correlations thus furnish an approximate quantitative window for the amount of AuCl_3_ incorporated during sequential doping.

Further optical characteristics, particularly the transitions at wavelengths over 1300 nm, were analyzed by Fourier‐transform infrared (FT‐IR) spectroscopy. Upon (bi)polaron formation, high‐, low‐polaron, and bipolaron bands appeared between the valence and conduction bands, indicating the presence of a bandgap. We focused on the appearance of peaks generated by (bi)polarons, located within the wavenumber range of 1600–4000 cm^−1^, and the infrared‐active vibrational (IRAV) mode below 1600 cm^−1^ (Figure 1c).^[^ ^23^, ^24^, ^25^ ^]^ The absorbance in the IRAV mode especially increased at higher dopant concentration, implying disruption of bond symmetry due to hole formation and charge creation.^[^ ^24^ ^]^ This observation confirmed that (bi)polarons were generated within the IDTBT backbone, consistent with the UV–vis–NIR results.

Electron spin resonance (ESR) analysis was employed to examine the charge carrier species and their conduction states, specifically focusing on the variation in the population of polarons with dopant concentration.^[^ ^26^, ^27^ ^]^ ESR analysis detects spins, including those originating from polarons (unpaired electrons) but not bipolarons (paired electrons). Analysis of the first‐derivative ESR spectra disclosed an increase in ESR signal intensities as [AuCl_3_] increased to 1 mM, suggesting an increase in the number of polarons within the doped IDTBT film (Figure 1d). A subsequent reduction in the ESR signal intensities was observed in IDTBT films doped with [AuCl_3_] in the range of 2–5 mM, demonstrating the partial conversion of polarons into bipolarons at relatively high dopant concentrations. At even higher dopant concentrations over 10 mM, the ESR peak intensity was observed to increase again, suggesting the generation of additional polarons (Figure S4, Supporting Information).

To quantify the polaron density in the doped IDTBT films based on ESR results, we calibrated the doped CP films using CuSO_4_·5H_2_O as a reference (Figure 1e).^[^ ^28^ ^]^ The polaron concentration in the undoped IDTBT film was measured as 2.86 × 10^18^ cm^−3^, which increased to 1.89 × 10^19^ cm^−3^ at [AuCl_3_] = 1 mM. The concentration then decreased to 1.12 × 10^19^ cm^−3^ at [AuCl_3_] = 5 mM and eventually increased slightly to 1.67 × 10^19^ cm^−3^ at [AuCl_3_] = 10 mM. This result aligned with the trend of the bipolaron absorption peaks at ≈1200 nm in the UV–vis–NIR absorption spectra of the IDTBT films doped with AuCl_3_. The ESR analysis results establish that the polaron concentration increases by ≈6.5‐fold upon doping with 1 mM AuCl_3_. At [AuCl_3_] ≥ 1 mM, the conversion rate of formed polarons into bipolarons surpasses the generation rate of new polarons, resulting in a temporary reduction in the overall polaron concentration. Interestingly, this turning point coincides with the occurrence of polarity switching.

### 2.2 Characterization of Polarity Switching in Electronics and Energy Perspectives

**Figure 2:**
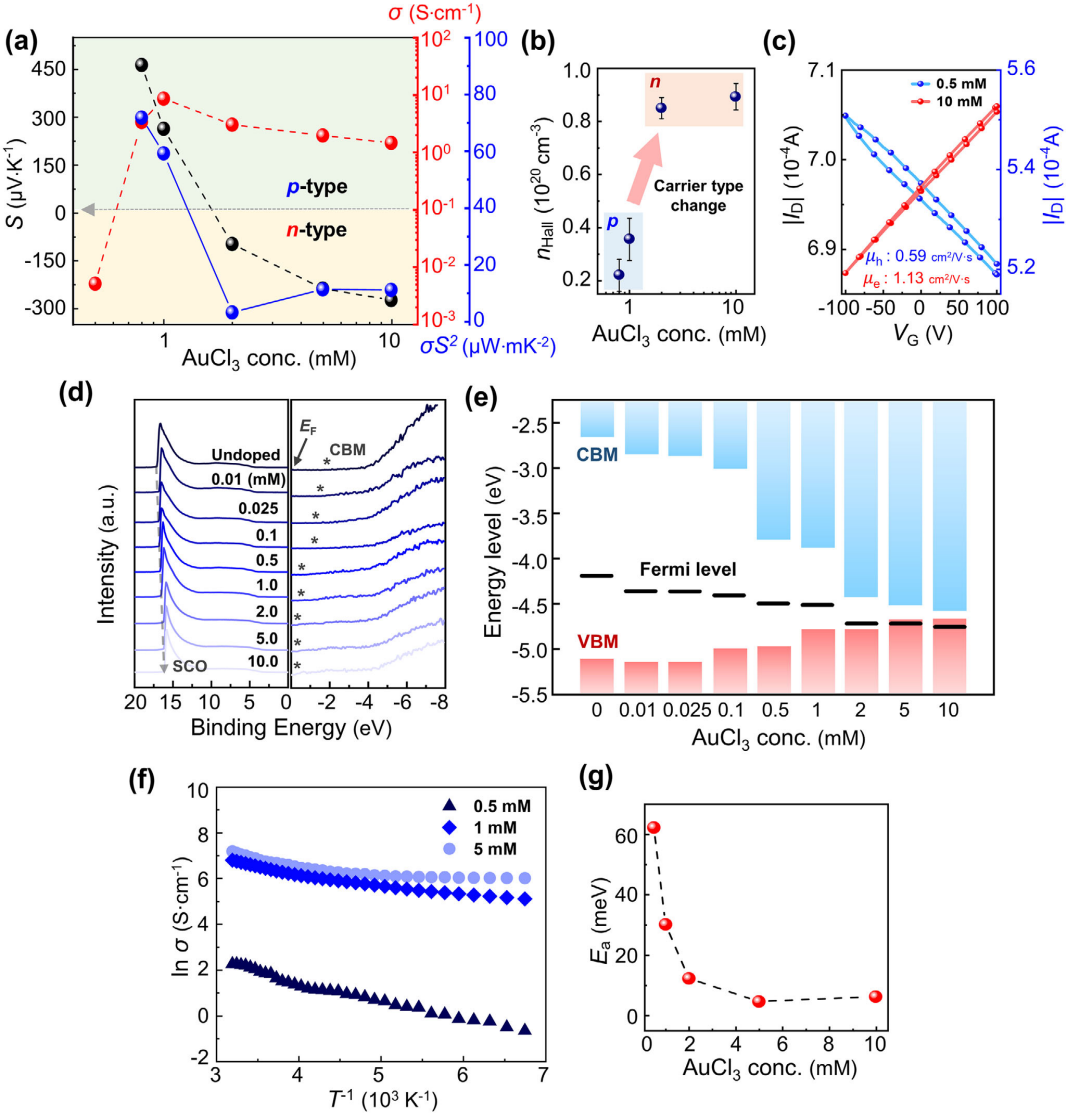
Charge transport properties and energy levels variation of doped IDTBT films. a) Seebeck coefficient (*S*), electrical conductivity (*σ*), and power factor (*σS* ^2^) trends of AuCl_3_‐doped IDTBT films. b) Hall carrier density *n*_Hall _as a function of dopant concentration. (c) Transfer characteristics of 4‐point probe FETs prepared from AuCl_3_‐doped IDTBT films before and after polarity switching. d) UPS (left) and IPES (right) spectra of doped IDTBT films and e) corresponding band diagrams. f) *T*‐dependent *σ* measurement results for Arrhenius plotting. (g) Activation energy *E*_a_ of corresponding films according to doping concentration.

In *p*‐type doping, holes serve as the primary charge carriers,^[^ ^22^ ^]^ leading to an increase in the electrical conductivity (*σ*) of IDTBT films. However, beyond a specific dopant concentration ([AuCl_3_] ≥ 2 mM), electrons become the primary carriers, leading to *n*‐type charge transport (**Figure** **2**a). The polarity of the majority of charge carriers in IDTBT can be determined by *S*, as its sign indicates the dominant charge carrier type.^[^ ^18^, ^29^ ^]^ Upon doping, *σ* increased while *S* decreased due to increased carrier concentration. After *σ* reached a maximum at [AuCl_3_] = 1 mM, a decline of σ occurred with a negative *S* value for [AuCl_3_] ≥ 2 mM. This result suggests that mobile electrons replace holes as the primary delocalized charge carriers in heavily doped IDTBT films, providing compelling evidence of polarity switching from *p‐* to *n‐*type.^[^ ^17^ ^]^ In addition, the power factor (PF = *S* ^2^ *σ*) decreased along with the *S* value until *S* transitioned to a negative value. After this transition, PF increased concomitant with the absolute value of *S*.

The nature of the charge carriers in the CP films was ascertained using AC Hall measurements.^[^ ^17^ ^]^ Only coherent carriers contribute to the Hall voltage because such carriers interact with the classic Lorentz force.^[^ ^30^ ^]^ By contrast, incoherent carriers, such as hopping carriers, are affected by the transverse Hall electric field, causing them to move in the direction opposite to that of the Lorentz force. This phenomenon results in a reduction of the Hall voltage.^[^ ^30^ ^]^ The emergence of delocalized charge carriers makes the Hall effect measurable, with more band‐like characteristics observed for doped IDTBT films. In our experiment, the AC Hall measurements were conducted in the dopant range of [AuCl_3_] = 0.8 to 10 mm. The drastic transition in carrier type from *p*‐ to *n*‐type was observed in the narrow [AuCl_3_] range of 1–2 mM (Figure 2b). This transition coincides with the [AuCl_3_] value where the sign of *S* changed from positive to negative (Table S4, Supporting Information). From these results, we infer that increasing numbers of coherent carriers were generated as the doping level increased, thereby making them the dominant charge carriers.

To observe the transport properties, including those of incoherent carriers, we evaluated the charge carrier mobility (*µ*) values in four point‐probe field‐effect transistors (FETs) (Figure 2c). This method involves applying an electric field to induce charge carrier drift, regardless of carrier coherency.^[^ ^31^ ^]^ The drain current (*I*_D_) of highly doped OSCs was modulated by controlling the gate voltage (*V*_G_) in the linear regime (drain voltage *V*_D_ = ± 0.5 V). The transfer curve of the undoped IDTBT film exhibited typical *p*‐type operation with an average hole *µ* of 1.1 ± 0.1 cm^2^ V^−1^ s^−1^ (Figure S5, Supporting Information).^[^ ^32^ ^]^ Doped IDTBT films exhibited surprisingly clear *n*‐type transfer characteristics at relatively high doping levels above 2 mM, despite IDTBT being originally known as a representative *p‐*type semiconductor. The *µ* values were found to be 0.59 ± 0.04 cm^2^ V^−1^ s^−1^ for holes at [AuCl_3_] = 0.5 mM and 1.13 ± 0.05 cm^2^ V^−1^ s^−1^ for electrons at [AuCl_3_] = 10 mM (Figure S6, Supporting Information). Again, polarity switching from *p*‐ to *n*‐type was observed in the highly doped IDTBT films.

To explain this polarity switching phenomenon, it is essential to understand the changes in electronic structure within the doped CP films. Cyclic voltammetry (CV) analysis revealed that undoped IDTBT has a lowest unoccupied molecular orbital (LUMO) level of −3.52 eV, a highest occupied molecular orbital (HOMO) level of −5.26 eV, and a band gap of 1.74 eV (Figure S7, Supporting Information).^[^ ^33^ ^]^ Through the combination of ultraviolet photoelectron spectroscopy (UPS)^[^ ^34^ ^]^ and inverse photoelectron spectroscopy (IPES),^[^ ^35^ ^]^ photoelectron spectroscopy (PES) analysis was used to elucidate the occupied and unoccupied energy states, providing insights into the conduction band minimum (CBM), valence band maximum (VBM), work function, and *E*_F_ of the doped IDTBT films^[^ ^36^ ^]^ (Figure S8, Supporting Information). Upon doping, numerous charge carriers populate the bandgap states, shifting the *E*_F_ closer to the conduction band. In our results, the *E*_F_ tended to move toward the valence band edge, whereas the work function exhibited an increasing trend (Figure 2d,e). Moreover, the CBM approached the *E*_F_ for [AuCl_3_] ≥ 5 mM. This shift enables easier access to the conduction band, creating an almost continuous electronic band, in turn leading to *n*‐type charge conduction. Given that increasing the dopant concentration causes the *E*_F_ to be extremely close to the CBM and the band gap to decrease, it is reasonable to conclude that the heavily doped IDTBT film transitions to metal‐like characteristics, with an almost closed band gap.

It is important to distinguish the observed *n*‐type charge transport from real metallic transport behaviors. The relationship between *σ* and temperature (*T*) was investigated by plotting 1000 *T* ^−1^ against ln (*σ*) (Figure 2f). The activation energy (*E*_a_) represents the energy barrier that charge carriers, bound to charge‐transfer complexes, need to overcome to be liberated as free carriers.^[^ ^37^ ^]^ *E*_a_ values were determined by calculating the slope of plots using the Arrhenius equation $\sigma =\sigma_{0}\;\cdot e^{\left(-\frac{E_{a}}{k_{B}T}\right)}$. In general, for hopping transport, *σ* increases with the thermal energy as more carriers gain the energy to overcome the *E*_a_ barrier.^[^ ^37^ ^]^ As the dopant concentration increased, the doped IDTBT films exhibited a reduction in the slope of the 1000*T* ^−1^‐ln (*σ*) graph, suggesting a decrease in *E*_a_ (Figure 2g; Figure S9a, Supporting Information). Notably, *E*_a_ became significantly lower above [AuCl_3_] = 5 mM. As the doping concentration increased, the negative slope value decreased significantly, but the slope did not become positive. However, at [AuCl_3_] = 10 mM, *σ* decreased as *T* increased from 233 to 323 K, showing metal‐like transport (Figure S9b, Supporting Information).

Although an ideal metallic behavior was not confirmed, the *σ* data could be effectively modeled using a general variable‐range hopping model, specifically $\sigma \;\left(T\right)=\sigma_{0}\;\;exp\left[-{\left(\frac{T_{0}}{T}\right)}^{\frac{1}{d}}\right]$, where *T*_0_ is the characteristic temperature.^[^ ^38^, ^39^ ^]^ Upon fitting, the *d* values were found to be ≈1 and 2 for [AuCl_3_] = 2 mm. The Efros–Shklovskii variable‐range‐hopping model (*d* = 2) was used to elucidate the soft Coulomb gap in the density‐of‐states (DOS) close to the *E*_F_, arising from Coulombic interactions between localized states.^[^ ^39^ ^]^ In addition, the *d* value was 3.9 for [AuCl_3_] = 5 mM, which is close to the *d* value of 4 corresponding to hopping transport in either 3D localized states or 2D doped interfacial states (Figure S9, Supporting Information). Despite challenges in elucidating the charge transport using a single model, the analysis of the *d* value is useful because it revealed the presence of coherent charge transport alongside hopping transport in the doped IDTBT films. Moreover, the work function increased and the surface potential decreased as the dopant concentration increased, as measured by Kelvin probe force microscopy (KPFM) (Figure S10, Supporting Information). These results are consistent with the UPS and IPES results that showed a decrease in both work function and band gap. Lowering the *E*_a_ by increasing the dopant concentration suggests the possibility of achieving coherent transport by excess doping as discussed earlier.

Next, to quantify the charge localization dynamics, we calculated the peak‐to‐peak distance from the second‐derivative ESR signal. According to the Elliot–Yafet mechanism, the peak‐to‐peak distance increases when initially isolated spin carriers become denser or when dopant‐induced scattering increases.^[^ ^40^ ^]^ Furthermore, this mechanism induces spin‐orbit coupling, leading to a reduction in the relaxation time, which is the interval before spin collisions.^[^ ^40^ ^]^ Therefore, the peak‐to‐peak distance serves as an indicator of the extent of spin localization. In our study, the calculated value remained constant for [AuCl_3_] ≤ 1 mM but increased for [AuCl_3_] ≥ 2 mM (Figure S11, Supporting Information). These results indicate that the polarons are primarily localized and isolated at low dopant concentrations, with minimal mutual interactions. As the dopant concentration increases, however, interactions among polarons become more pronounced and lead to a gradual reduction in their localized and isolated nature. For [AuCl_3_] ≤ 1 mM, the carrier spins demonstrated clear localization and isolation. This behavior diminished significantly at [AuCl_3_] ≥ 2 mM, indicating a transition to *n*‐type charge transport. Overall, these findings reveal that excess doping of IDTBT not only induces polarity switching but promotes a greater degree of charge carrier delocalization, resulting in more coherent charge transport.

### 2.3 Doping‐Induced Microstructural Changes

**Figure 3:**
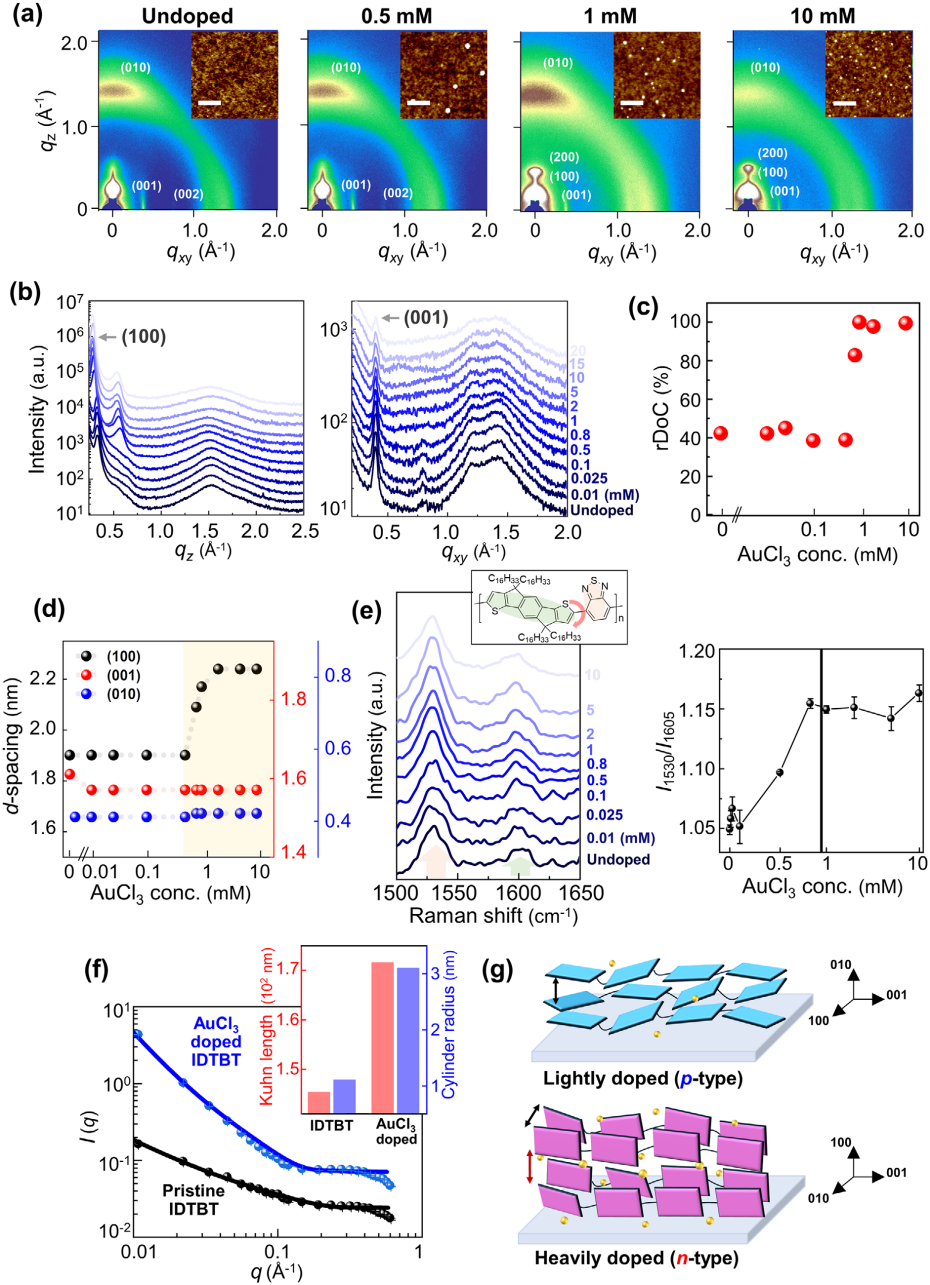
Microstructural analysis of IDTBT films with controlled AuCl_3_ dopant concentration. a) 2D GI‐WAXS patterns of AuCl_3_‐doped IDTBT films. The insets show corresponding AFM images, and the scale bars are 0.5 µm. b) Out‐of‐plane and in‐plane 1D profiles of the GI‐WAXS patterns. The relative degree of crystallinity (rDoC) c) and *d*‐spacing values d) as a function of AuCl_3_ concentration. e) Raman spectra (left) and relative scattering intensity ratio (*I*_1530_/*I*_1605_, right) of AuCl_3_‐doped IDTBT films. The inset indicates the vibrational position at IDTBT backbones related to the Raman spectra. f) Fitted SANS profiles of pristine and AuCl_3_‐doped IDTBTs. The inset shows extracted Kuhn length and cylinder radius values. g) Schemes of microstructure change from lightly doped (*p*‐type) to heavily doped (*n*‐type) IDTBT films.

Polarity switching induced by the introduction of bulky dopant materials may inevitably result in nanostructural and morphological changes in doped CP films. To assess this, we examined the surface morphologies of both undoped and doped IDTBT films using atomic force microscopy (AFM) (insets of **Figure** **3**a). As the dopant concentration was increased from 0 to 10 mm, a slight increase in the surface roughness from 30 to 55 nm was noted, accompanied by a higher presence of Au nanoparticles (tiny white spots) within the same observation range. We also observed that as the doping concentration increases, the average size of the Au nanoparticles decreases (from ≈100 nm at 1 mM, to ≈40–50 nm at 2 mM, and ≈20–30 nm at 10 mM), while their number density increases accordingly (Figure S12, Supporting Information).

Grazing incidence wide‐angle X‐ray scattering (GI‐WAXS) analysis was conducted to gather information on the crystalline structure of the doped films.^[^ ^41^ ^]^ The diffraction pattern of pristine IDTBT film is characterized by the presence of the (010) peak (*q* ∼ 1.5 Å^−1^) for the out‐of‐plane (*q*_z_) direction and the (001) peak (*q* ∼ 0.4 Å^−1^) and (002) peak (*q* ∼ 0.8 Å^−1^) for the in‐plane (*q*_xy_) direction (Figure S13, Supporting Information).^[^ ^19^, ^42^, ^43^ ^]^ Notably, the pristine IDTBT film exhibits relatively strong out‐of‐plane (010) and in‐plane (00l) peaks, indicatives of a face‐on dominant texture (Figure 3a). Upon doping, the lamellar stacking distance in the *q*_z_ direction increased from 1.9 to 2.24 nm through incorporation of AuCl_3_ (molecular size ≈0.3 nm) (Table S5, Supporting Information). This increase in lamellar distance suggests that the dopants preferentially infiltrate the side chains of the IDTBT molecules rather than inserting in between the IDTBT backbones.^[^ ^44^, ^45^ ^]^ The out‐of‐plane *q*_z_ (200) peak emerged clearly at [AuCl_3_] = 0.8 mM, and its intensity gradually increased as the dopant concentration was increased above that value (Figure 3b). Simultaneously, the *q*_xy_ (002) peak was steadily diminished, and *q*_z_ (010) peaks were intensified while the *π–π* stacking distance remained nearly constant. Generally, *q*_z_ (*h*00) and *q*_xy_ (010) peaks represent the edge‐on orientation of IDTBT crystals.^[^ ^46^ ^]^ Therefore, the pronounced evolution of the *q*_z_ (200) peak suggests that doping induces a transition from amorphous to crystalline structure, with a more edge‐on‐dominant orientation. The driving force behind this dominant orientational change may stem from the additional charges accumulated on the CP backbones.^[^ ^47^ ^]^ These accumulated charges would generate strong forces that lead to reorganizing the chains, thereby optimizing CP backbone packing and stabilizing the overall CP film structure.

To quantitatively confirm the increased crystallinity suggested by the above results, the GI‐WAXS diffraction pattern was integrated over an azimuthal angle range of 0 (in‐plane) to 90° (out‐of‐plane) to obtain the relative degree of crystallinity (rDoC). Compared to the undoped sample, the rDoC increased more than twofold as the dopant concentration increased beyond 0.8 mM. This marked increase in rDoC can be attributed to dopant‐induced ordering (Figure 3c),^[^ ^4^ ^]^ a relatively rare phenomenon in doped materials because the incorporation of dopants typically reduces crystallinity significantly. Furthermore, the observed increase in the lamellar distance of the out‐of‐plane (100) peak, coupled with almost unchanged backbone (001) and *π–π* stacking (010) distances, suggests that the introduced dopants predominantly infiltrate the side chains along the (*h*00) direction, rather than affecting the *π–π* stacking or inter‐backbone spacing (Figure 3d). To summarize, AuCl_3_ penetrates the lamellar stacking, causing (re‐)ordering of the backbones through either electrostatic interactions or straightforward physical insertion between side chains.

To further characterize the structural changes in the heavily doped IDTBT films, Raman spectroscopic analysis was conducted, focusing on the peaks at 1605 and 1530 cm^−1^ associated with the stretching modes of the IDT‐ and BT‐ rings, respectively (Figure 3e). The planarity of the IDTBT backbone was determined by calculating the peak‐intensity ratio (*I*_1530_/*I*_1605_).^[^ ^48^, ^49^ ^]^ This ratio is inversely related to the torsional angle between the IDT and BT units, as a smaller angle reflects increased backbone planarity. Enhanced planarity strengthens *π*‐conjugation and facilitates electron delocalization, which in turn amplifies the Raman activity of the BT unit—leading to a relative increase in the 1530 cm^−1^ peak intensity compared to the 1605 cm^−1^ peak. The peak ratio exhibited a significant increase for [AuCl_3_] ≥ 0.5 mM, indicating the onset of electrical effects induced by the dopant ions that became saturated at higher dopant concentrations. The planarity of the doped IDTBT backbones significantly increased when the dopant concentration exceeded a threshold of [AuCl_3_] = 0.8 mM. This result is consistent with an increase in chain rigidity explained by an increase in Kuhn length. Solution‐based small‐angle Neutron Scattering (SANS) analysis revealed an increase in the Kuhn length after doping, rising from 145.3 nm in the undoped state to 171.6 nm in the AuCl_3_‐doped state (Figure 3f; Figure S14, Supporting Information).^[^ ^50^ ^]^ The results of the Raman spectroscopy and solution SANS analysis indicate that doping increases the planarity of the IDTBT backbone, supporting the GI‐WAXS findings showing an increase in the rDoC. At low doping levels, dopants will preferentially diffuse into the relatively loose amorphous regions rather than the densely packed crystalline domains that have begun to form.^[^ ^19^ ^]^ However, as the crystallinity increases and more crystals form, the amount of dopants infiltrating the lamellar regions of edge‐on crystals would increase. In this process, the backbone ordering would become slightly more planar because the dopant ions mainly infiltrate along the lamellar direction, which would not significantly disrupt the backbone order but rather enhance crystallization in a reinforcing manner.

Angle‐dependent near‐edge X‐ray absorption fine structure (NEXAFS) analysis was performed to support our inferences regarding thin‐film microstructural change, specifically focusing on the average orientation of the conjugated backbone planes in both the crystalline and amorphous regions of the IDTBT films (Figure S15, Supporting Information). The undoped IDTBT film exhibited predominant face‐on thin film properties. The dichroic ratio *R*‐value, which reflects the orientation of conjugated planes, changed slightly upon doping.^[^ ^51^, ^52^ ^]^ Specifically, the face‐on orientation of the original undoped IDTBT film (*R* = −0.64) decreased markedly at [AuCl_3_] = 10 mM (*R* = −0.38). This reduction in the *R‐*value suggested a doping‐induced decrease in the face‐on texture, supporting the transformation from face‐on to edge‐on, consistent with the GI‐WAXS results (Figure 3b,c)

The structural information obtained from the various analyses, including multiscale structural changes from meso‐ to nanoscale, is summarized schematically in Figure 3g. As the dopant concentration increased beyond 0.8 mM, the rDoC increased twofold, transitioning its plane orientation from predominantly face‐on to edge‐on. The separation between lamellar stacking increased upon the introduction of dopants, whereas the *d‐*spacing of the *π–π* stacking remained almost unchanged. The doping‐induced enhancement of the crystallinity is expected to provide well‐ordered charge transport pathways, reducing obstacles and promoting charge movements.^[^ ^4^ ^]^ The edge‐on alignment also facilitates lateral charge transport and enhances the *σ* of doped IDTBT samples through efficient *π–π* stacking interactions.^[^ ^11^ ^]^ Additionally, building on the characteristic intramolecular charge transport along the backbone of IDTBT,^[^ ^49^ ^]^ the increase in Kuhn length and chain planarity is expected to further enhance charge transport along the backbone. Based on the collective results, the polarity switching observed when the dopant concentration becomes sufficiently high appears to occur when a structural change is preceded by the stiffening of the chain and a shift in backbone orientation, followed by the introduction of excess dopants.

### 2.4 Polarity Switching Mechanism

**Figure 4:**
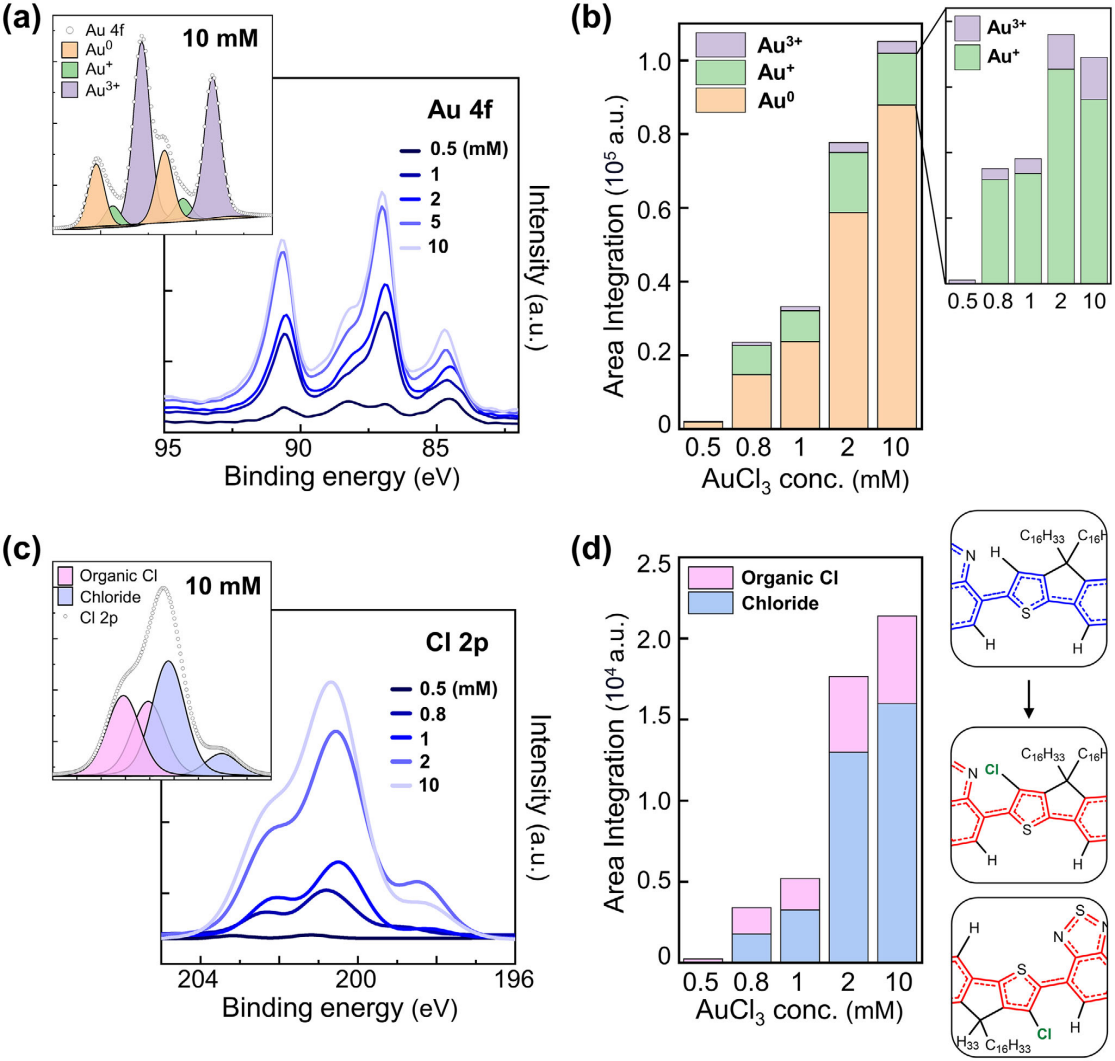
a) Au 4f XPS spectra of IDTBT films doped with AuCl_3_. The inset shows a spectrum of an IDTBT film doped with 10 mm AuCl_3_. b) Area integration analysis of Au^0^, Au^+^, and Au^3+^ peaks. The inset shows enlarged Au^+^ and Au^3+^ portions. c) Cl 2p XPS spectra of IDTBT films doped with AuCl_3_. d) Area integration analysis of Cl 2p peaks according to the AuCl_3_ concentration and expected C···Cl positions with the chemical structure from *p*‐doped IDTBT (blue‐colored, right top) to *n*‐doped IDTBT (red‐colored, right bottom).

It is essential to establish the mechanism underlying the polarity switching observed at high dopant concentrations. The mechanism underlying *p‐*type doping between CP and AuCl_3_ was delineated previously.^[^ ^53^, ^54^ ^]^ However, understanding of this phenomenon from a chemical perspective remains poor because the doping process involves complicated interactions among the CP, dopant ions, and doping byproducts. To gain insight into the interactions and chemical states of these materials, elemental analysis of the doped CP films was systematically conducted using X‐ray photoelectron spectroscopy (XPS), leading to examining changes in binding energies, oxidation states, and the relative ratios of ionic elements. In the S 2p spectrum, oxidation of the thiophene group in IDTBT led to red‐shifts of the two characteristic peaks at 164 and 165 eV upon doping (Figure S16a, Supporting Information). Additionally, a lowering of the C 1s binding energy was also observed (Figure S16b, Supporting Information).^[^ ^55^ ^]^ Next we focused on the Au and Cl peaks to monitor the status of the dopant. From the leftmost peak in the XPS profile of Au 4f, Au^3+^4f^5/2^, Au^+^ 4f^5/2^, Au^0^ 4f^5/2^, Au^3+^ 4f^7/2^, Au^+^ 4f^7/2^, and Au^0^ 4f^7/2^ were observed in the binding energy range of 82–94 eV (**Figure** **4**a; Figure S16c, Supporting Information).^[^ ^53^, ^56^ ^]^ As [AuCl_3_] was increased from 0.5 to 10 mM, a progressive rise in Au 4f binding energy was observed, accompanied by a steady enhancement of the relative intensity of each deconvoluted Au peak. The relative ratios of the deconvoluted peaks provide information that can be used to propose the chemical mechanism underlying the doping process.

In the typical Lewis acid doping process, the AuCl_3_ that has diffused into the polymer matrix is converted to AuCl_2_^⁻^ and AuCl_4_^⁻^ by accepting an electron from the IDTBT backbone, resulting in [IDTBT]⁺ and *p‐*type doping behavior. However, when the dopant concentration becomes sufficiently high that AuCl_2_^⁻^ is present in excess in the reaction environment (i.e., within the polymer matrix), some AuCl_2_^⁻^ may precipitate to the more stable Au^0^. Simultaneously, reactions involving AuCl_4_^⁻^ and Cl^⁻^ can also take place.^[^ ^53^, ^54^ ^]^ The Au(I)Cl_2_⁻ species generated during the redox reaction between IDTBT and AuCl_3_ is likely to undergo, ligand‐assisted disproportionation to Au^0^ and Au(III) within the sulfur‐incorporating IDT environment, thereby rendering the second‐line equation in the proposed reaction scheme chemically plausible.^[^ ^57^ ^]^ These processes are expected to occur at higher dopant concentrations within the solid‐state CP film. AuCl_4_^⁻^, AuCl_2_^⁻^, and Cl^⁻^ serve as counterions to [IDTBT]⁺ for stabilization. Given that AuCl_4_^⁻^ is a large ion that tends to form ion clusters, the presence of excessive dopants will lead to strong electrostatic repulsion, also promoting some degree of Cl decomposition. This entire process can be summarized as follows with detailed explanation (Note S1, Supporting Information):

(1) $$2\mathrm{IDTBT}+2\mathrm{AuC}l_{3}\to 2{\left[\mathrm{IDTBT}\right]}^{+}+{\left[\mathrm{AuC}l_{2}\right]}^{-}+{\left[\mathrm{AuC}l_{4}\right]}^{-}$$

(2) $$3{\left[\mathrm{AuC}l_{2}\right]}^{-}\to 2Au^{0}↓+{\left[\mathrm{AuC}l_{4}\right]}^{-}+2Cl^{-}$$

(3) $${\left[\mathrm{IDTBT}\right]}^{+}+{\left[\mathrm{Au}\left(\mathrm{III}\right)Cl_{4}\right]}^{-}+2Cl^{-}\to \left[\mathrm{IDTB}T^{+}-2\mathrm{Cl}\right]+{\left[\mathrm{Au}\left(I\right)Cl_{2}\right]}^{-}$$

This mechanism can be verified by deconvoluting the XPS peaks to determine the forms in which each element exists. In particular, the Au 4f peak was analyzed in greater detail, as illustrated in a bar graph (Figure 4b). Integration of each peak provided an estimation of the relative quantity of the corresponding bonds. Among the Au species, Au° constitutes the largest proportion. Au^0^ is the most stable form of Au, precipitating as solid nanoparticles after Au^3+^ accepts three electrons during the doping process.^[^ ^53^, ^54^ ^]^ Since Au^0^ is the final product of doping, the continuous increase in Au^0^ suggests that the doping process is ongoing. Next, the composition ratio of Au ions follows the order of Au^+^ then Au^3+^. Although it was expected that the ratios of both of these ions would increase steadily as doping progressed, a decrease in the Au⁺ proportion was observed at AuCl_3_ concentrations above 2 mM. This result suggests that [AuCl_2_]^⁻^ is converted to Au^0^, [AuCl_4_]^⁻^, and Cl^⁻^ during the *p‐n* switching process. Given the presence of Cl⁻ in the mechanism, it is essential to determine where the Cl ultimately ends up. By examining the deconvoluted Cl 2p XPS spectrum, four Cl 2p_3/2_ and Cl 2p_1/2_ peaks can be categorized into chloride peaks at 198 and 201 eV and organic Cl peaks at 202 and 203 eV (Figure 4c).^[^ ^58^ ^]^ According to the analysis, the intensity of the chloride peak increased consistently upon doping as more and more dopant molecules gradually entered, with the organic Cl peak also showing a significant increase (Figure 4d). This result demonstrates the formation of new C─Cl bonds, indicating that Cl has bonded to the IDTBT backbone. Specifically, [IDTBT]^•+^ reacts with Cl^⁻^ ions to form [IDTBT‐Cl]^•⁻^ in the presence of excess AuCl_3_.^[^ ^59^ ^]^ As the IDTBT backbone transitions into a quinoid structure under doping, the thiophene ring, which is positively charged, will provide the most energetically stable position for doping‐induced chlorination.^[^ ^48^, ^60^, ^61^ ^]^ To corroborate this hypothesis, we subjected the thin film to harsh environmental conditions to induce dedoping. Subsequent UV‐vis‐NIR spectral analysis revealed a blue shift in the neutral peak after dedoping process, which we attribute to chlorine substitution at the IDT moiety. The chlorine substituent acts as an electron‐withdrawing group; through its inductive effect it induces a blue shift in the IDTBT backbone, as evidenced by the spectral transition (Figure S17, Supporting Information). To substantiate chlorine substitution, we conducted solution‐state ¹H NMR and Time‐Dependent Density Functional Theory (TD‐DFT) analyses (Figures S18 and S19–S22, Supporting Information). The NMR spectrum shows complete disappearance of the aromatic signal at ≈8 ppm, indicating chemical modification. TD‐DFT calculations reveal that the ICT (*π*–π*) and neutral transitions of chlorine‐substituted structures differ from those of chlorine addition, but with systematic blue shifts: ICT maxima at 410.8, 441.7, 431.9, and 461.6 nm and neutral peaks at 653.9, 710.4, 670.7, and 779.6 nm for IDT‐Cl(1)‐BT, IDT‐Cl(2)‐BT, IDT‐Cl(3)‐BT, and IDTBT‐Cl, respectively. The magnitude of the shift follows the order IDT‐Cl(1)‐BT > IDT‐Cl(3)‐BT > IDT‐Cl(2)‐BT > IDTBT‐Cl, matching the pronounced blue shifts observed in heavily doped films where chlorine occupies the IDT‐Cl(1) and IDT‐Cl(3) positions. Furthermore, regarding the possibility of alternative mechanisms, such as the formation of [Au(I)Cl_2_]⁻ complexes anchored to the thiophene sulfur atoms, we performed sulfur XPS measurements on films doped with 10 mM AuCl_3_. In addition to the original thiophene peak (164 eV), we observed the emergence of a thiolate peak (161 eV), suggesting the formation of covalent S–Au bonds and possible anchoring of AuCl_2_^⁻^ to the IDTBT backbone (Figure S23, Supporting Information). While this observation supports the existence of such interactions, it remains unclear whether this anchoring plays a critical role in the observed polarity switching. If S–Au anchoring was the primary mechanism, it would be difficult to reconcile with the absence of polarity switching in P3HT or the FeCl_3_‐induced polarity switching in DPP‐type polymers. Therefore, additional studies are necessary to clarify the precise function of such coordination complexes during polarity switching. In this process, the attached Cl^⁻^ or AuCl_2_^⁻^ ion may deliver its negative charge to the IDTBT backbone, thereby spreading the transferred electrons. The charges thus delocalize along the *π‐*conjugated backbone and act as a carrier for *n*‐type charge transport. To validate the universal applicability of the aforementioned electrochemical redox reaction, we systematically introduced diverse dopants into IDTBT and applied AuCl_3_ to alternative CP systems. Remarkably, IDTBT exhibited *p‐n* polarity switching not only with AuCl_3_ but also with AuBr_3_ and VCl_3_. A mechanistic rationale for this behavior is detailed in Note S2 (Supporting Information). Here, the existing positive (bi)polaron would help compensate for the negative charge.^[^ ^17^ ^]^ Another possibility is that gold ions chelate with functional groups in the polymer backbone, further enhancing stabilization.^[^ ^62^, ^63^ ^]^ However, the exact identity of the counter‐cation responsible for stabilizing the negative polaron remains unclear.

Importantly, the first evidence of structural changes in the doped CPs was observed at a doping concentration of ≈0.8 mM, while the polarity shift was observed within the range of 1–2 mM. The discrepancy between the onset of structural and polarity changes suggests that, while the doping‐induced structural rearrangements may contribute to the polarity shift, they are not necessarily the direct cause. The current findings thus suggest a more complex interplay between these two phenomena rather than a simple causal relationship. Excessive doping led to a significant increase in overall charge concentration, which in turn resulted in stronger electrostatic interactions akin to those observed in ionic crystals.^[^ ^64^, ^65^, ^66^ ^]^ This effect enhanced the crystallinity and improved the molecular ordering, as evidenced by the changes in orientation and ordering observed in Figure 3d and Figure S15 (Supporting Information). The observed structural reorganization is reminiscent of the formation of cocrystals, where donor and acceptor molecules co‐assemble into highly ordered lattices.^[^ ^64^, ^65^, ^66^ ^]^ Such improved ordering and orientation would contribute to the enhanced charge transport characteristics of the doped system, reinforcing the idea that doping not only introduces charge carriers but promotes favorable structural transformations that facilitate charge conduction.

### 2.5 Application of Polarity Switching to Devices

The exceptional environmental stability of *n‐*type CP thin films achieved through polarity switching makes them promising candidates for *p‐n* diode applications. However, CP‐based *p‐n* diodes have historically demonstrated poor rectification ratios compared to their inorganic counterparts, particularly in devices utilizing a single doping system for bipolar charge transport.^[^ ^7^, ^12^, ^67^ ^]^ This performance gap originates from challenges associated with achieving precise control over dopant profiles and interfacial properties in CP‐based electronics. The polarity switched CP films reported here overcome these constraints, demonstrating outstanding environmental and thermal stability, and satisfying the demands of high‐performance electronic devices. UV–vis–NIR spectroscopy of the doped IDTBT films conducted at various temperatures revealed negligible spectral changes after 240 h at 25 °C (Figure S24a–f, Supporting Information), underscoring their exceptional resistance to environmental degradation. Furthermore, the doped IDTBT films maintained their polaron and bipolaron absorption peaks after 12 h at 70 °C (Figure S24b, Supporting Information) and even after exposure to 120 °C for 4 h (Figure S24c, Supporting Information). The *S* value of the polarity switched IDTBT film remainted negative even after 7 days at 25 °C (Figure S24f, Supporting Information). These results demonstrate the viability of polarity switched AuCl_3_‐doped films for use in stable and efficient devices intended for *n*‐type charge conduction.

**Figure 5:**
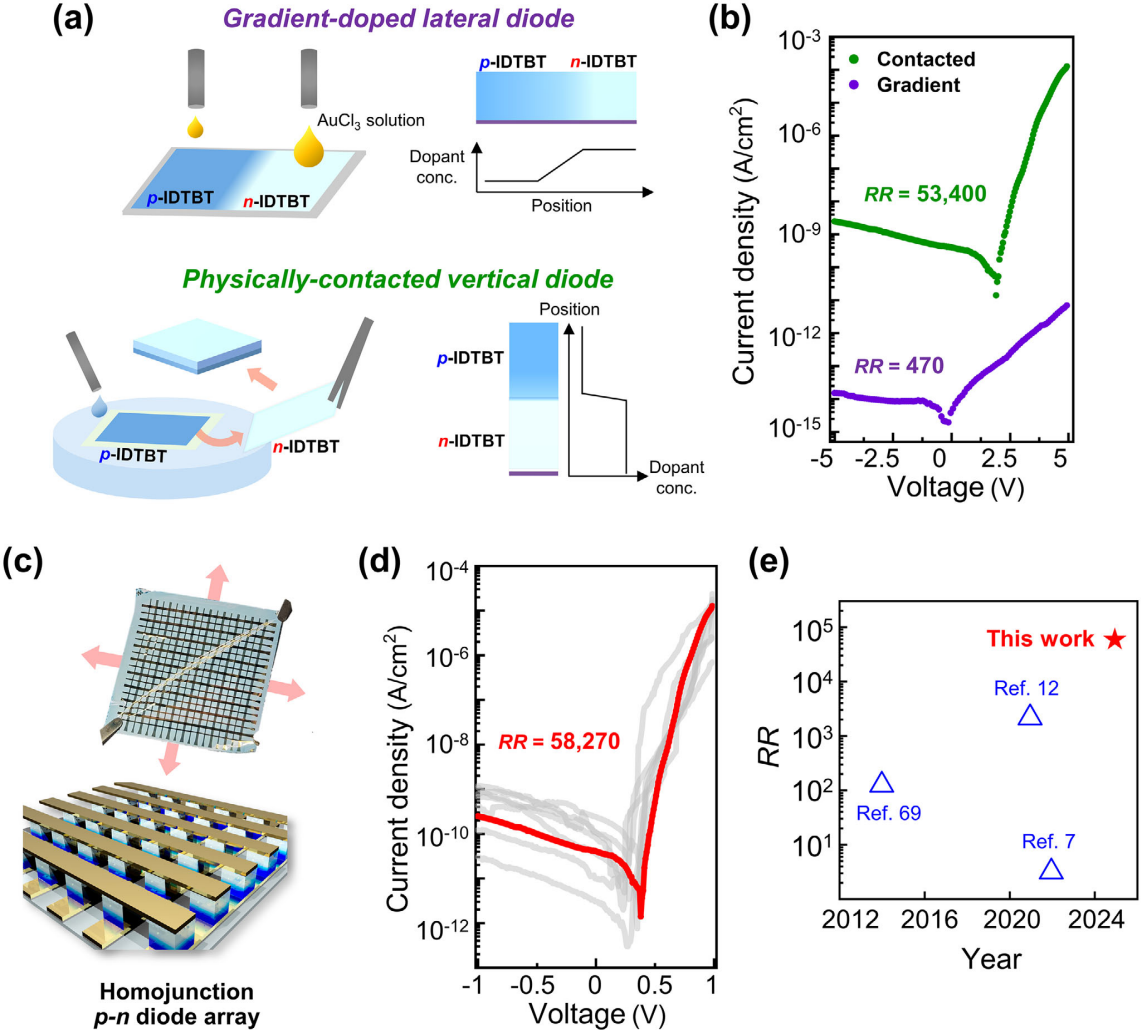
a) Schemes of gradient‐doped lateral (top) and physically‐contacted vertical homojunction *p‐n* diodes (bottom). b) *J–* *V* curves of gradient‐doped (rectification ratio, *RR* = ≈470) and physically‐contacted (*RR* = ≈53400) homojunction *p‐n* diodes. c) Scheme and picture of large‐area (two‐inch sized) physically‐contacted homojunction *p‐n* diode array. d) *J*–*V* curves of *p‐n* diode array with an averaged *RR* of ≈58270 (red line). e) A comparison of *RR* values with reported organic homojunction diodes.^[^ ^7^, ^12^, ^69^ ^]^

To further demonstrate the usefulness of the *n*‐switched CP films, we fabricated homojunction *p‐n* diodes consisting of only AuCl_3_‐doped IDTBT films. The electrical characteristics of lateral and vertical diode structures were compared (**Figure** **5**a). The lateral‐type devices were prepared by naturally graded AuCl_3_ doping (namely, a gradient‐doped homojunction), while the vertical devices were constructed by stamping a freestanding *p*‐doped IDTBT film with an *n*‐doped IDTBT film (namely, a physically contacted homojunction). The measured current density–voltage (*J–V*) characteristics demonstrated that the physically contacted homojunction diodes had larger rectification ratios of up to ≈53400 compared to those of the gradient‐doped homojunction devices (≈470) (Figure 5b). We attribute that gradient‐doped homojunction produces a non‐abrupt, rougher interfacial profile characterized by spatially gradual carrier concentration changes. This diffuse homojunction leads to a broader depletion region, a less pronounced built‐in potential, and weaker electric field distribution—all of which diminish the barrier to reverse carrier injection. (Figure S25, Supporting Information). Furthermore, a diode array was fabricated to confirm that it can be run multiple times on different channel dimensions (Figure 5c). The large‐area array device on a two‐inch die successfully yielded a well‐formed homojunction array with an average rectification ratio of 58270, showing superior performance to previously reported homojunction structures based on CP materials (Figure 5d; Table S6, Supporting Information). Furthermore, the ln(*J*)–ln(*V*) curve obtained from the current characteristics of the standard diode reveals three distinct regions:, i.e., the space‐charge effect region, the trap‐filled limit region, and the trap‐free region (Figure S26, Supporting Information). These results confirm the feasibility of high‐performance organic homojunction rectifying diodes and highlight their potential for use in scalable, reliable organic electronic devices.

## 3 Conclusion

We successfully achieved polarity switching by doping an IDTBT film with an excess amount of AuCl_3_, an oxidizing dopant. The IDTBT film exhibited ordinary *p*‐type charge transport when undoped and lightly doped, but transitioned to *n*‐type charge transport when [AuCl_3_] reached 2 mM. This transition was validated by the conversion of the Seebeck coefficient from positive to negative, showing a charge transport type transition in four point‐probe FETs and AC Hall measurements. As the doping concentration increased, the generation of additional charges and progressive filling of electronic states led to more coherent and delocalized charge transport. This was accompanied by a significant reduction in the transport gap and bandgap, allowing the heavily doped IDTBT film to exhibit metal‐like charge transport characteristics. Furthermore, the introduction of charged dopant ions induced notable structural changes in both crystalline and amorphous regions, including the development of a predominantly edge‐on orientation and enhanced rDoC within the crystalline domains. These structural modifications played a crucial role in facilitating the efficient transport of coherent charge carriers. The evolving chemical interactions between the CP and the dopant during the doping process were investigated through XPS, with a particular focus on the role of Au and Cl from the dopant ions. Leveraging the polarity switching phenomenon, we successfully fabricated a homojunction *p*‐*n* diode array by utilizing a single CP‐dopant system for both the *p*‐ and *n*‐type layers, resulting in a rectification ratio of 10^4^–10^5^. This proof‐of‐concept demonstrates the viability of utilizing a single CP‐dopant system in CP‐based devices. Ultimately, this study reveals the chemical mechanism underlying polarity switching accompanied by structural changes. These findings pave the way for innovative advancements in CP‐based electronics and sustainable energy technologies.

## 4 Experimental Section

### Materials

IDTBT ($\bar{M_{W}}$/$\bar{M_{n}}$ = 250k/100k) was purchased from Derthon Optoelectronic Materials Science and Technology Co. Ltd. AuCl_3_, which was used as the dopant in this system, was purchased from Sigma–Aldrich. In addition, chloroform and acetonitrile, which were used to dissolve IDTBT and AuCl_3_, respectively, were purchased from Sigma–Aldrich. All products were used without further purification.

### Film Preparation

An IDTBT solution (5 mg mL^−1^) was prepared by dissolving IDTBT in chloroform. As‐doped Si/SiO_2_ (3000 Å, *C*_i_ = 1.08 × 10^−8^ F cm^−2^) substrates were cleaned using an Extran/deionized water/acetone/isopropyl alcohol mixture and treated with an octadecyl trichlorosilane solution diluted in toluene. Each film was spin‐coated using the IDTBT solution and thermally annealed at 483K for 30 min. Sequential doping was performed using an AuCl_3_ solution by dissolving AuCl_3_ in acetonitrile (Sigma Aldrich, anhydrous 99%). Each coated film was immersed in AuCl_3_ solutions of different concentrations (0.01–20 mM) for 3 min at room temperature.

### Device Fabrication

Top‐gate bottom‐contact and bottom‐gate top‐contact field‐effect transistors were fabricated for FET mobility measurements. Each device was based on an As‐doped Si/SiO_2_ (3000 Å, *C*_i_ = 1.08 × 10^−8^ F cm^−2^) substrate. The former device used a Ti (5 nm)/Au (15 nm) source and drain electrodes. The channel length and width were 100 µm and 1 mm, respectively. After the active‐layer coating and chemical doping, the CYTOP‐809 M (AGC Inc.) diluent was spin‐coated onto the substrates to produce a dielectric layer with a thickness of 500–600 nm. Al (50 nm) was used as the gate electrode. The latter device used an Au (50 nm) source and drain electrodes. Gradient‐doped lateral (Si/SiO_2_/*p, n*‐IDTBT/Au) and physically‐contacted vertical (Ti/Au/*n*‐IDTBT/*p*‐IDTBT/Au) homojunction *p*‐*n* diodes were fabricated. *p*‐IDTBT was coated on the PEDOT:PSS‐coated glass substrate and PEDOT:PSS was used as sacrificial layer for physically‐contacted device fabrication process. In addition, the *p‐n* diode array was fabricated vertically (Ti/Au/*n*‐IDTBT/*p*‐IDTBT/Au). The optimum channel length was between 0.25 and 0.5 mm. After the CP solution was spin‐coated on the substrate, the *n*‐IDTBT layer was chemically doped, and the *p*‐IDTBT layer was transferred onto the *n*‐IDTBT layer. The channel length and area were ≈1000 µm and 4.5 × 10^−7^ m^2^, respectively. All electrical characteristics were measured using a Keithley 4200SCS semiconductor parameter characterization system with a vacuum chamber probe station (MS‐TECH).

### Characterization

UV–vis–NIR absorption spectroscopy was performed using a V‐770 spectrometer (Jasco). The absorption spectra were recorded in the wavelength region of 300–2500 nm at intervals of 0.5 nm. The film samples were prepared on bare glass substrates, and a baseline was set with a transparent glass substrate. FT‐IR spectra were obtained using a Bruker VERTEX 70 instrument with polymer films under vacuum conditions. The spectra were measured in the transmittance mode in the wavenumber range of 400–4000 cm^−1^. Solution SANS measurements were conducted using a 40 m U‐SANS. CP was diluted by deuterated chloroform with a concentration of 5 mg mL^−1^. Neutrons with a wavelength of 0.6 nm were employed, with two sample‐to‐detector distances (4.7 m and 1.16 m) used to cover a *q* range from 0.01 to 0.6 Å^−1^. The recorded scattering intensities were adjusted to account for background scattering, scattering from the empty cell, and the sensitivity of individual detector pixels. The scattering data was then fitted by SasView software using the flexible cylinder with the power law model. AFM and Kelvin probe force microscopy were performed using an NX 10 (Park Systems) in the tapping and EFM modes for AFM (Tip: OMCL‐AC160TS) and KPFM (Tip: NSC36/Cr‐Au), respectively. GI‐WAXS and NEXAFS data were obtained at the 3C SAXS‐I, 9A U‐SAXS, 4D PES, and 10A2 HRPES beamlines of the Pohang Accelerator Laboratory (PAL, PLS‐II). All GI‐WAXS data were processed using the *p‐*GIXS program.^[^ ^68^ ^]^ AC Hall measurements were performed using a Lakeshore 8400. UPS and XPS were conducted using a Thermo Scientific ESCALAB 250Xi instrument with a He–I ultraviolet radiation source (21.2 eV). The *E*_F_ of all data was equilibrated with a pure Au sample. Raman spectra were obtained using an Alpha300R (Witec) with a 532‐nm excitation laser source. The *S* was calculated using laboratory‐made equipment composed of an Agilent 24700a, a Keithley 2400, a pair of Peltier modules, and copper/constantan thermocouples. JES‐X320 and JES‐FA100 (JEOL) ESR spectrometers were used for the ESR measurements. Low‐energy IPES was conducted using Versa Probe (ULVAC‐PHI Inc., Japan) equipped with an electron source. The near‐ultraviolet photons emitted from the sample were collected using a photon detector, including a bandpass filter with a central wavelength of 260 nm. The energy reference was calibrated using clean Ag. The base pressure of the analysis chamber was maintained at 2.0 × 10^−7^ Pa during the measurements. ^1^H NMR spectra (600 MHz) were recorded on a Bruker AVANCE III spectrometer with the samples dissolved in deuterated solvent.

### DFT Calculation

Geometry optimization was conducted using Avogadro and Gaussian 16 W software. The density functional B3LYP/6‐31G and BPW91/LANL2DZ basis sets were used. UV–vis–NIR spectra calculation was conducted TD‐DFT simulations using IDTBT dimers with the Time‐Dependent Franck–Condon (TD‐FC) basis set.

## Conflict of Interest

The authors declare no conflict of interest.

## Supporting information



Supporting Information

## Data Availability

The data that support the findings of this study are available from the corresponding author upon reasonable request.
