# Supplementary material for: Accompanying Structural Transformations in Polarity Switching of Heavily Doped Conjugated Polymers
Source: Adv Mater. 2025 Jul 9;37(39):2505945. doi: 10.1002/adma.202505945 (PMC12506605; doi:10.1002/adma.202505945)
Supplement: Supplementary file 1 — Supporting Information [file ADMA-37-2505945-s001.docx]

**Supporting Information for**

Accompanying Structural Transformations in Polarity Switching

of Heavily Doped Conjugated Polymers

*Eunsol Ok^,a,^*^†^*, Sein Chung^a^*^,†^*, Seung Hyun Kim^a^,* *Kitae Kim^b,c^, Soohyung Park^c^*, *Hoimin Kim^d^, Yeonjin Yi^b^, Jong Dae Jang^e^, Hansol Lee^f^,* *Hyun Ho Choi^g^, Boseok Kang^d,h,^* and Kilwon Cho^a,^**

^a^ Department of Chemical Engineering, Pohang University of Science and Technology, Pohang 37673, Republic of Korea

^b^ Department of Physics, Yonsei University, 50 Yonsei-ro, Seodaemun-gu, Seoul 03722, Republic of Korea

^c^ Division of Nano & Information Technology, KIST School, University of Science and Technology (UST), Seoul 02792, Republic of Korea

^d^ SKKU Advanced Institute of Nanotechnology (SAINT) and Department of Nano Science and Engineering, Sungkyunkwan University (SKKU), Suwon 16419, Republic of Korea

^e^ Neutron Science Division, Korea Atomic Energy Research Institute, 1045 Daedeok-daero, Yuseong-gu, Daejeon, 34057, Republic of Korea

^f^ School of Chemical, Biological and Battery Engineering, Gachon University, Seongnam 13120, Republic of Korea

^g^ Department of Materials Engineering and Convergence Technology, Gyeongsang National University, Jinju 52828, Republic of Korea

^h^ Department of Nano Engineering and Department of Semiconductor Convergence Engineering, Sungkyunkwan University (SKKU), Suwon 16419, Republic of Korea

*e-mail: bskang88@skku.edu (bk) and kwcho@postech.ac.kr (kc)

**Table of Contents**

**Supplementary Figures**1

**Fig. S1** Changes in the (a) color observed by optical microscope and (b) thickness of undoped and doped IDTBT films during sequential doping.1

**Fig. S2** Neutral peak intensity of UV-vis-NIR spectrum data of undoped and doped IDTBT films with controlled AuCl_3_ dopant concentration. 2

**Fig. S3** Solution UV-vis-NIR absorption spectra of mixed dual-solution system between IDTBT solution in chloroform and AuCl_3_ solution in acetonitrile with controlled weight percentage.3

**Fig. S4** ESR spectra of undoped and doped IDTBT films and the ESR signal intensity.4

**Fig. S5** FET transfer curve of undoped IDTBT films.5

**Fig. S6** FET transfer curves of doped IDTBT films in the linear regime.6

**Fig. S7** (a) Cyclic voltammetry (CV) analysis of undoped IDTBT films. (b) HOMO and LUMO levels of IDTBT film calculated by CV and DFT calculation (c) DFT calculation result of IDTBT monomer, dimer, and tetramer chains.7

Note 7

**Fig. S8** (a) Energy level information can be obtained by UPS and IPES. (b) Energy level diagrams of undoped and doped IDTBT films with controlled AuCl_3_ dopant concentration.8

**Fig. S9** (a) Temperature dependence of electrical conductivity and VRH model fitting of AuCl_3_ doped IDTBT. (b) Temperature dependence of electrical conductivity of 10 mM doped IDTBT.9

**Fig. S10** (a) KPFM surface profile images, (b) surface potential and work function data of undoped and doped IDTBT films with controlled AuCl_3_ dopant concentration.10

**Fig. S11** (a) ESR 2^nd^ derivative spectra. (b) Calculated peak-to-peak distance of undoped and doped IDTBT films with controlled AuCl_3_ dopant concentration.11

**Fig. S12** AFM height images of undoped and doped IDTBT films with controlled AuCl_3_ dopant concentration.12

**Fig. S13** 2D GI-WAXS patterns of undoped and doped IDTBT films with controlled AuCl_3_ dopant concentration.13

**Fig. S14** (a) SANS experiment geometry. (b) SANS evaluation metrics of pristine IDTBT solution and AuCl_3_ doped IDTBT solution.14

**Fig. S15** (a) Angle-dependent NEXAFS results and (b) *R* values of undoped and doped IDTBT films with controlled AuCl_3_ dopant concentration.15

**Fig. S16** (a) C 1s and (b) S 2p XPS spectra of undoped and AuCl_3_-doped IDTBT films. (c) Au 4f XPS spectra with deconvoluted peaks of IDTBT films with controlled AuCl_3_ dopant concentration. (d) Cl 2p XPS spectra with deconvoluted peaks for Cl 2p_3/2_ and Cl 2p_1/2_ peaks. 16

**Fig. S17** (a) Photographs of AuCl_3_-doped IDTBT thin-film dedoping processes with dry thermal annealing under harsh condition (b) UV-vis-NIR spectra for doped- (left) and after dedoped- (right) IDTBT thin films. 17

**Fig. S18** XPS spectra of AuCl_3_ doped- (4 graphs (C, S, Cl, and Au) on left side) and dedoped- (4 graphs (C, S, Cl, and Au) on right side) IDTBT thin films.18

**Fig. S19** Absorbance changes of UV-vis-NIR spectrum of AuCl_3_ 5 mM doped IDTBT films over time at (a) 25 ℃ (b) 70 ℃ (c) 120 ℃ (d) 150 ℃ (e) 180 ℃. (f) Seebeck coefficient (*S*), electrical conductivity (*σ*), and power factor (*σS*^2^) trends of AuCl_3_ 5 mM doped IDTBT films over time at 25 ℃.19

**Fig. S20** Lateral organic homojunction *p-n* diode fabricated from gradient-doped IDTBT.20

**Fig. S21** ln(*V*)-ln(*J*) plot of *p*-*n* diode data.21

Note 21

**Fig. S22** Preparation of AuBr_3_ solutions in ACN at various concentrations (mM) and sequential doping of IDTBT thin films, showing the color changes of both the IDTBT films and AuBr_3_ solutions after doping. (top) UV-vis-NIR spectra of IDTBT thin films doped with AuBr_3_ and their thermoelectric properties of the doped films, including the *p-n* polarity switching.22

**Fig. S23** UV-vis-NIR spectra of IDTBT thin films sequentially doped with VCl_3_ and their thermoelectric properties of the doped films, including the *p-n* polarity switching.23

**Fig. S24** UV-vis-NIR spectra of IDTBT thin films sequentially doped with FeCl_3_ and their thermoelectric properties of the doped films, including the *p-n* polarity switching.24

**Fig. S25** UV-vis-NIR spectra of PDPP-DTT and -4T thin films sequentially doped with AuCl_3_.25

**Supplementary Notes** 26

**Note S1** Electrochemical redox chemistry under AuCl_3_ doping process.26

**Note S2** Universality of polarity switching through heavy AuCl_3_ doping.27

**Supplementary Tables** 28

**Table S1** Reported other studies in which regard the Seebeck coefficient inversion or polarity switching.28

**Table S2** Reported other studies in which regard the chlorination usages from metal halides dopants for various nano- and conjugated materials.29

**Table S3** Concentration unit conversion table from mM to wt. % unit.30

**Table S4** AC Hall measurement parameters doped IDTBT films with controlled AuCl_3_ dopant concentration.31

**Table S5** GI-WAXS diffraction *q* values and calculated *d*-spacing data in (a) in-plane and (b) out-of-plane direction peaks of undoped and doped IDTBT films with controlled AuCl_3_ dopant concentration.32

**Table S6** A comparison of *RR* values with reported organic homojunction diodes using conjugated polymers.33

**Supplementary Reference** 34

**Supplementary Figures**


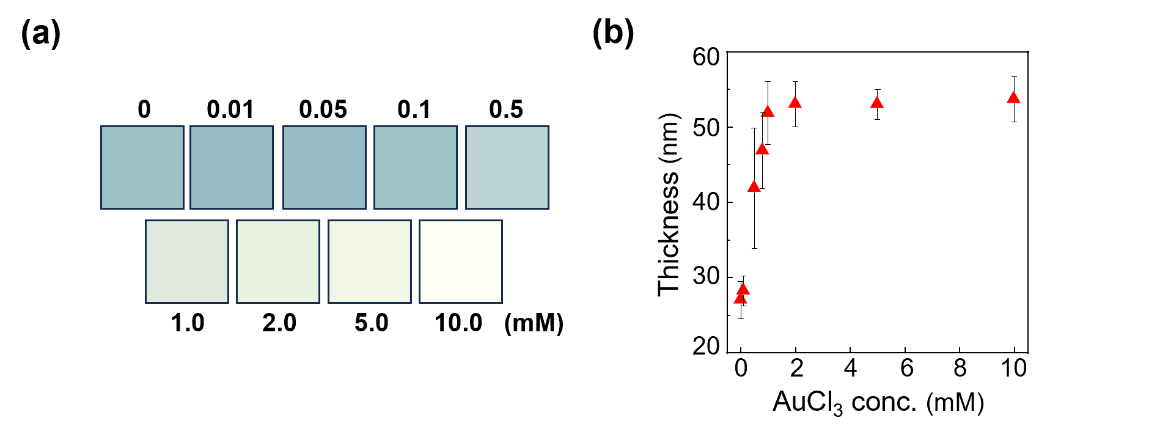


**Figure S1**. Changes in the (a) color observed by optical microscope and (b) thickness of undoped and doped IDTBT films during sequential doping.


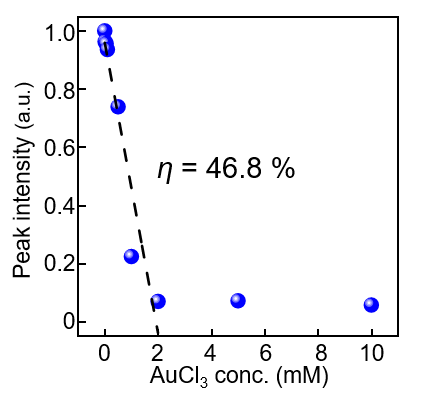


**Figure S2**. Neutral peak intensity of UV-vis-NIR spectrum data of undoped and doped IDTBT films with controlled AuCl_3_ dopant concentration. *η* is a slope of neutral peak intensity.


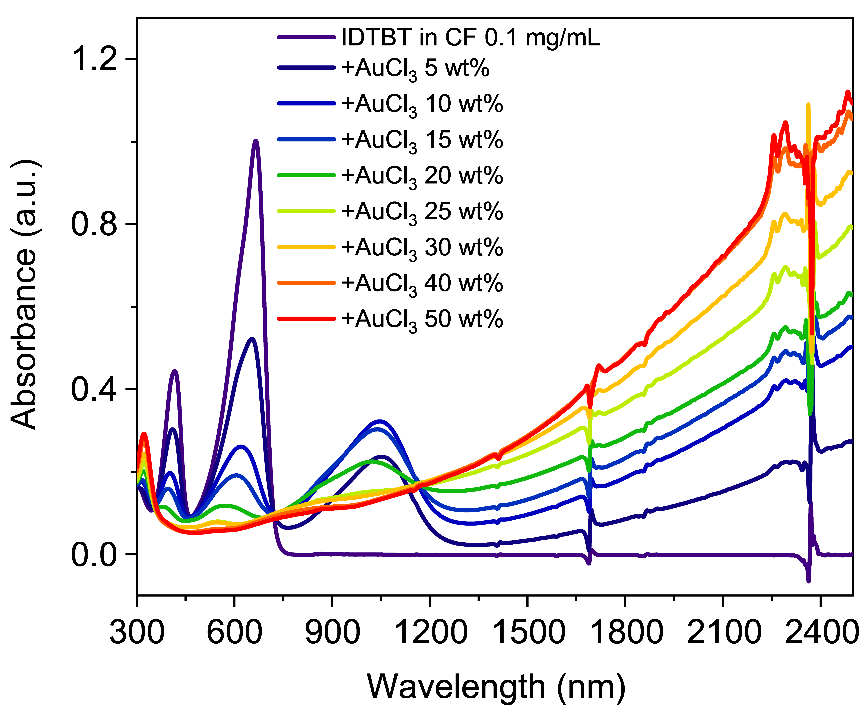

**Figure S3**. Solution UV-vis-NIR absorption spectra of mixed dual-solution system between IDTBT solution in chloroform (0.1 mg/mL) and AuCl_3_ solution in acetonitrile (1 mg/mL) with controlled weight percentage.


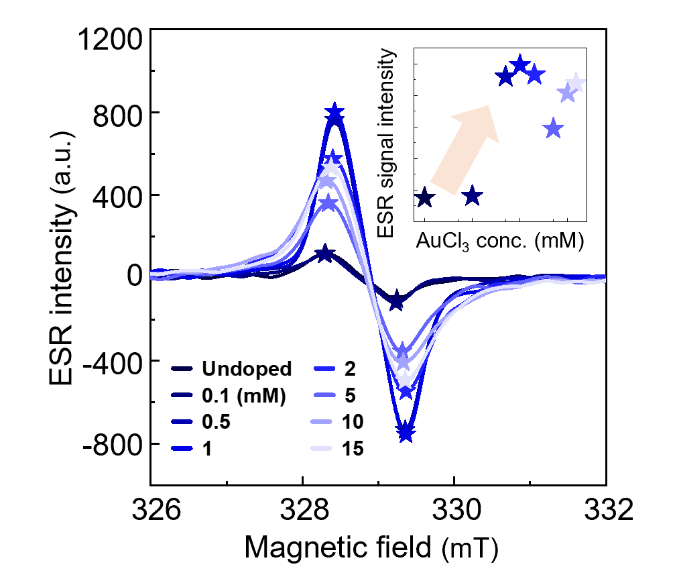


**Figure S4**. ESR spectra of undoped and doped IDTBT films and the ESR signal intensity (inset). Each intensity was calculated by integrating the area of ESR intensity curves and corrected by the thickness of each film.


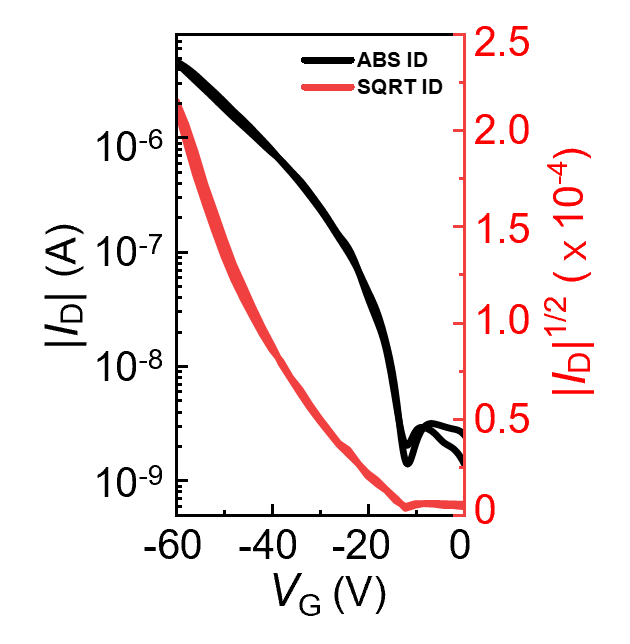


**Figure S5**. FET transfer curve of undoped IDTBT films. Average mobility ($\mu$_avg_), threshold voltage (*V*_th_), and on/off current ratio of the corresponding curves are 1.11 ± 0.1 cm^2^/V^.^s, -33.3 ± 0.3 V, and 3^.^10^4^.


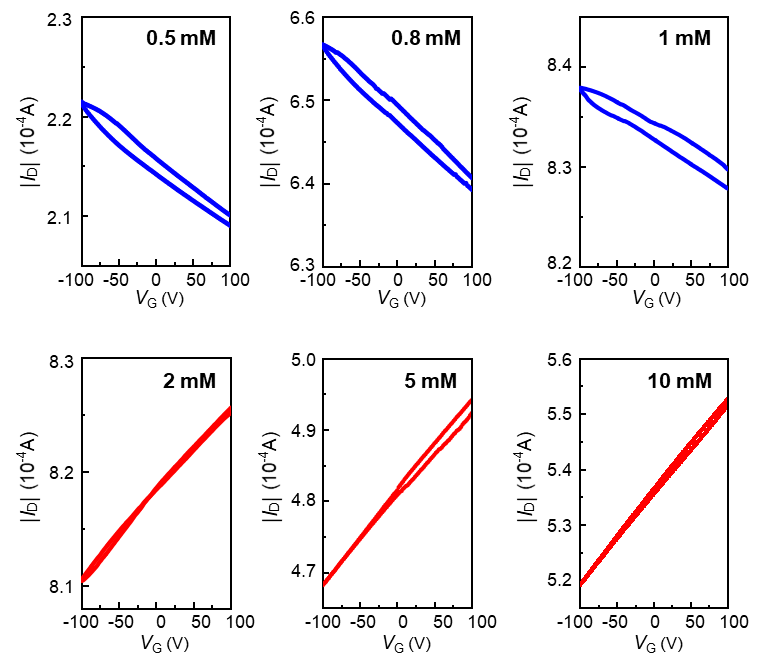


**Figure S6**. FET transfer curves of doped IDTBT films in the linear regime. |*V*_D_| = 0.5 V, *V*_G_ = -100 ~ 100 V.


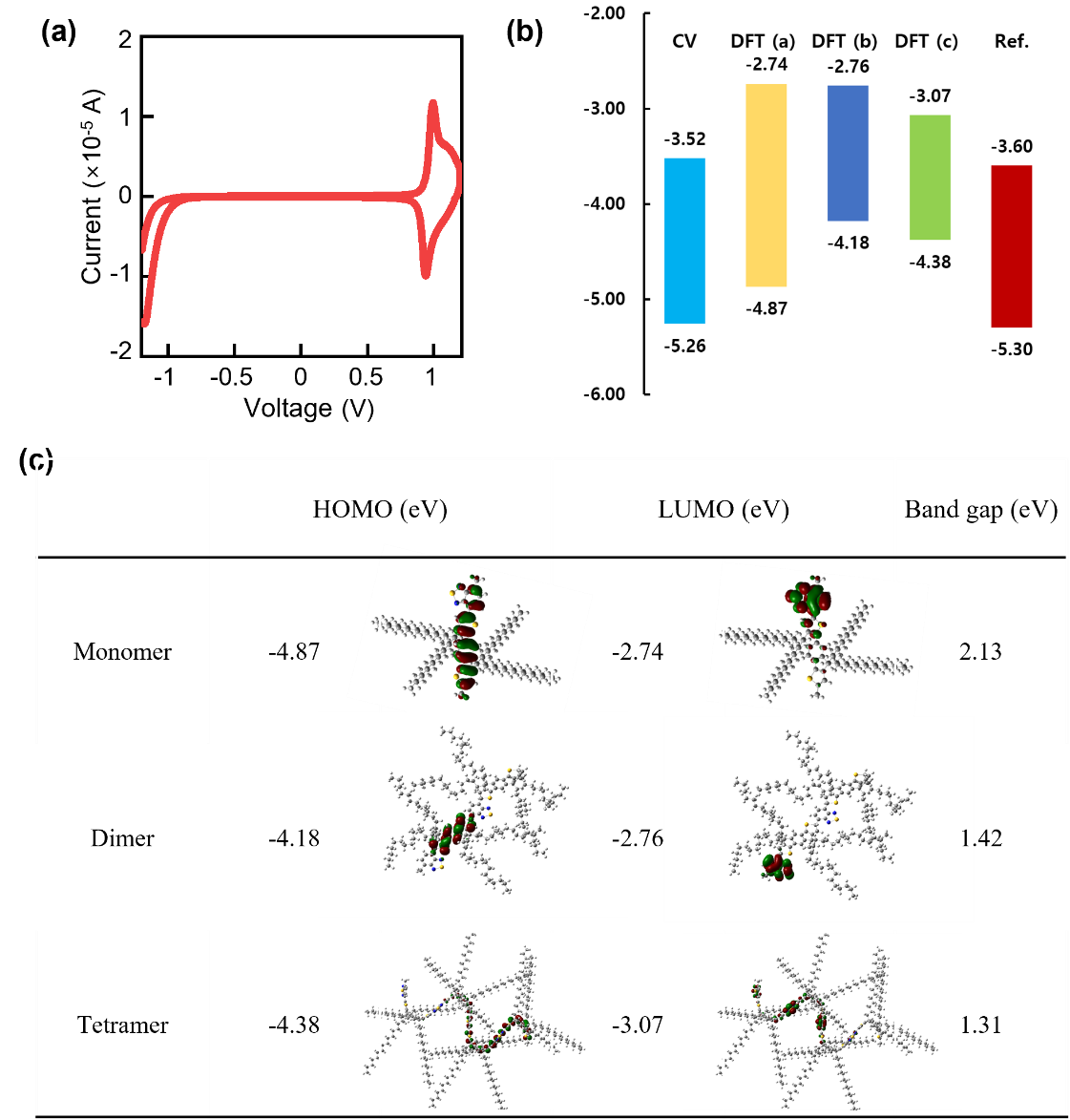


**Figure S7**. (a) Cyclic voltammetry (CV) analysis of undoped IDTBT films. The CP film was coated on ITO glass. Ferrocene was used as a reference. (b) HOMO and LUMO levels of IDTBT film calculated by CV and DFT calculation (IDTBT Neutral, dimer, no side chain) compared to reference level.^[S1]^ (c) DFT calculation result of IDTBT monomer, dimer, and tetramer chains. HOMO, LUMO level (eV), and band gap were calculated.

**Note:** The PES results indicate that the LUMO level of undoped IDTBT is -2.7 eV, differing by approximately 0.9 eV from the previously reported value of -3.60 eV. To ensure accuracy, precise measurements were performed using cyclic voltammetry, yielding a value of -3.52 eV, which matches the reported value. This discrepancy is thus attributed to the inherent limitations of the IPES technique in measuring vacant LUMO states.


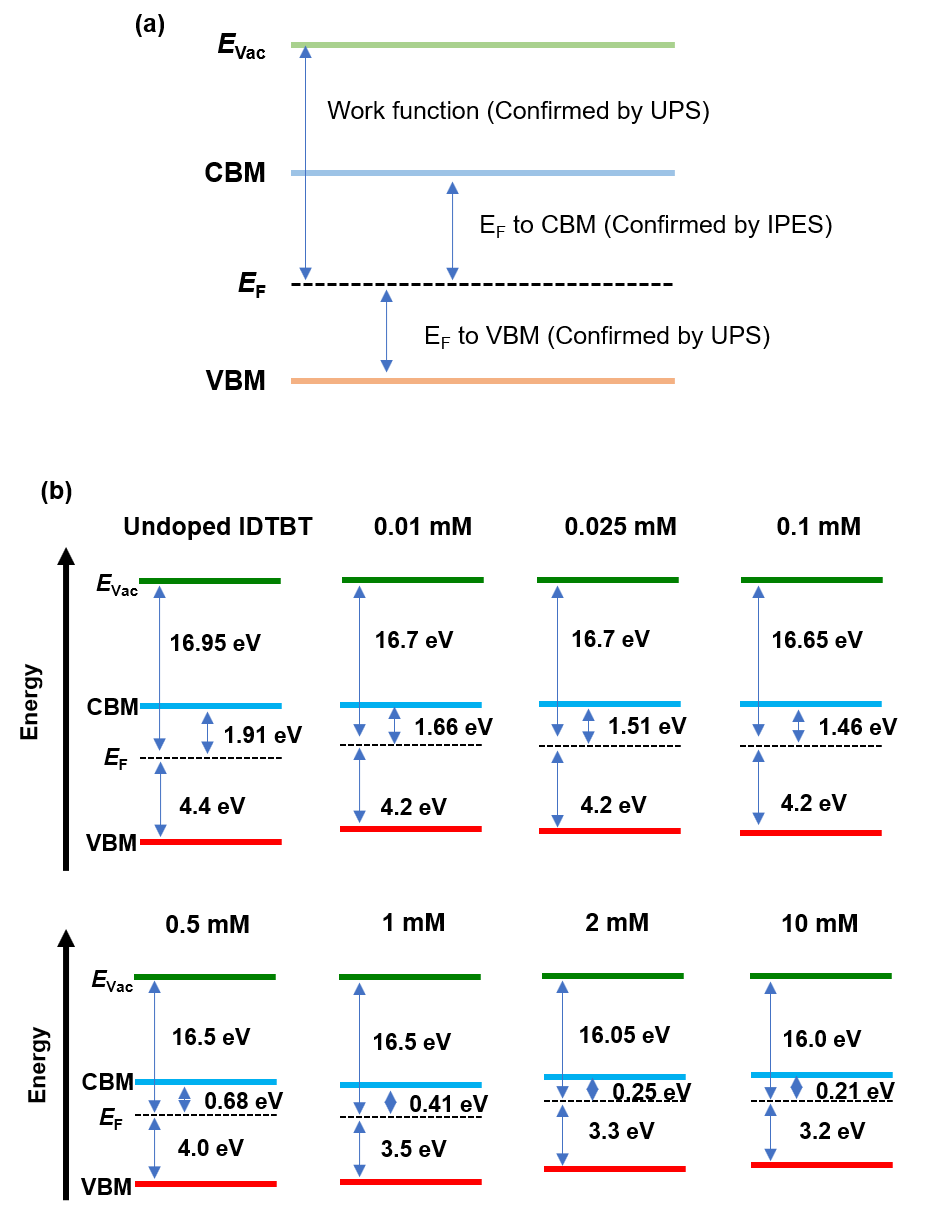


**Figure S8**. (a) Energy level information can be obtained by UPS and IPES. (b) Energy level diagrams of undoped and doped IDTBT films with controlled AuCl_3_ dopant concentration.
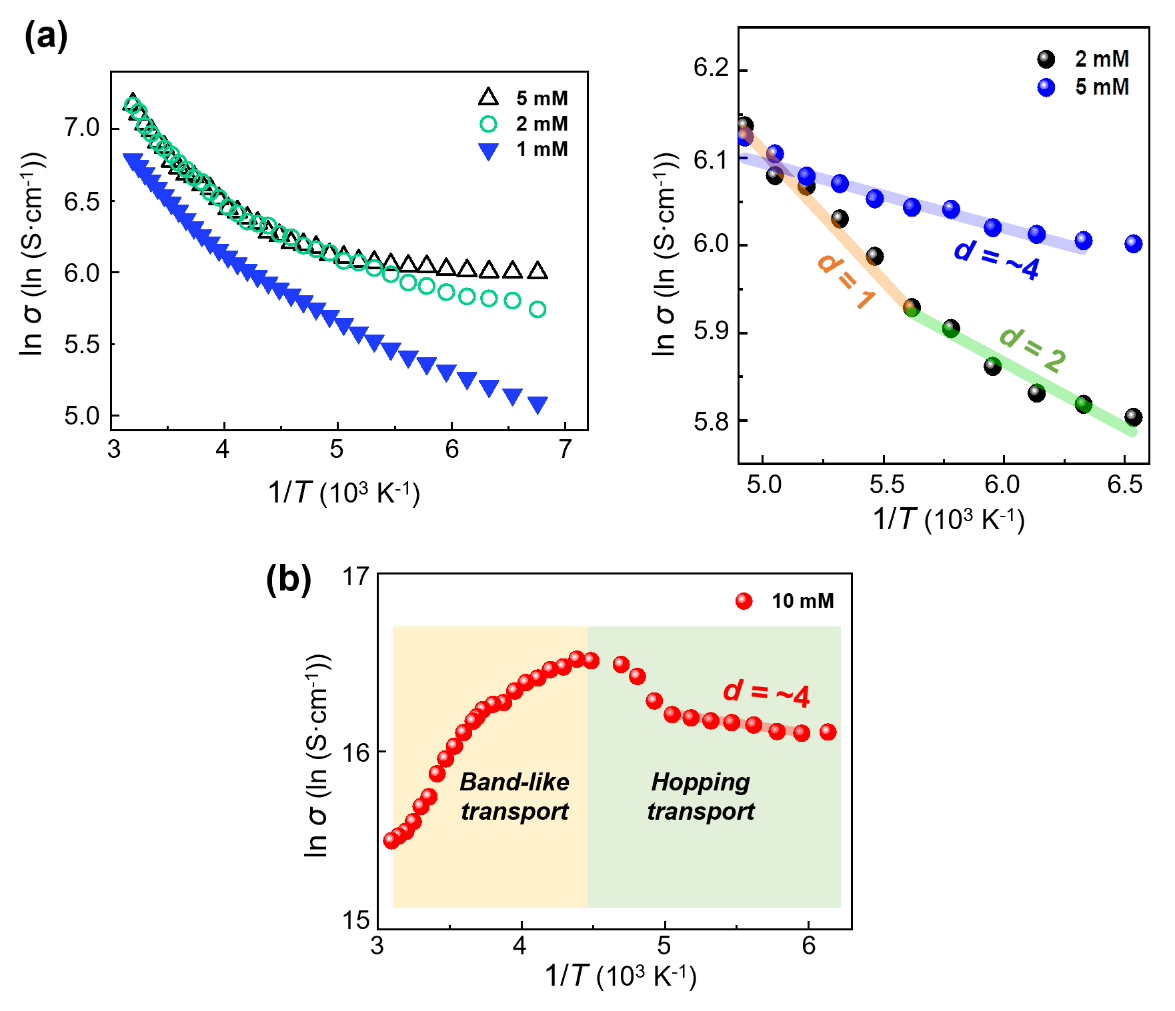


**Figure S9**. (a) Temperature dependence of electrical conductivity and VRH model fitting of AuCl_3_ doped IDTBT. Colors are set to distinguish the data of overlapped regions. (b) Temperature dependence of electrical conductivity of 10 mM doped IDTBT.


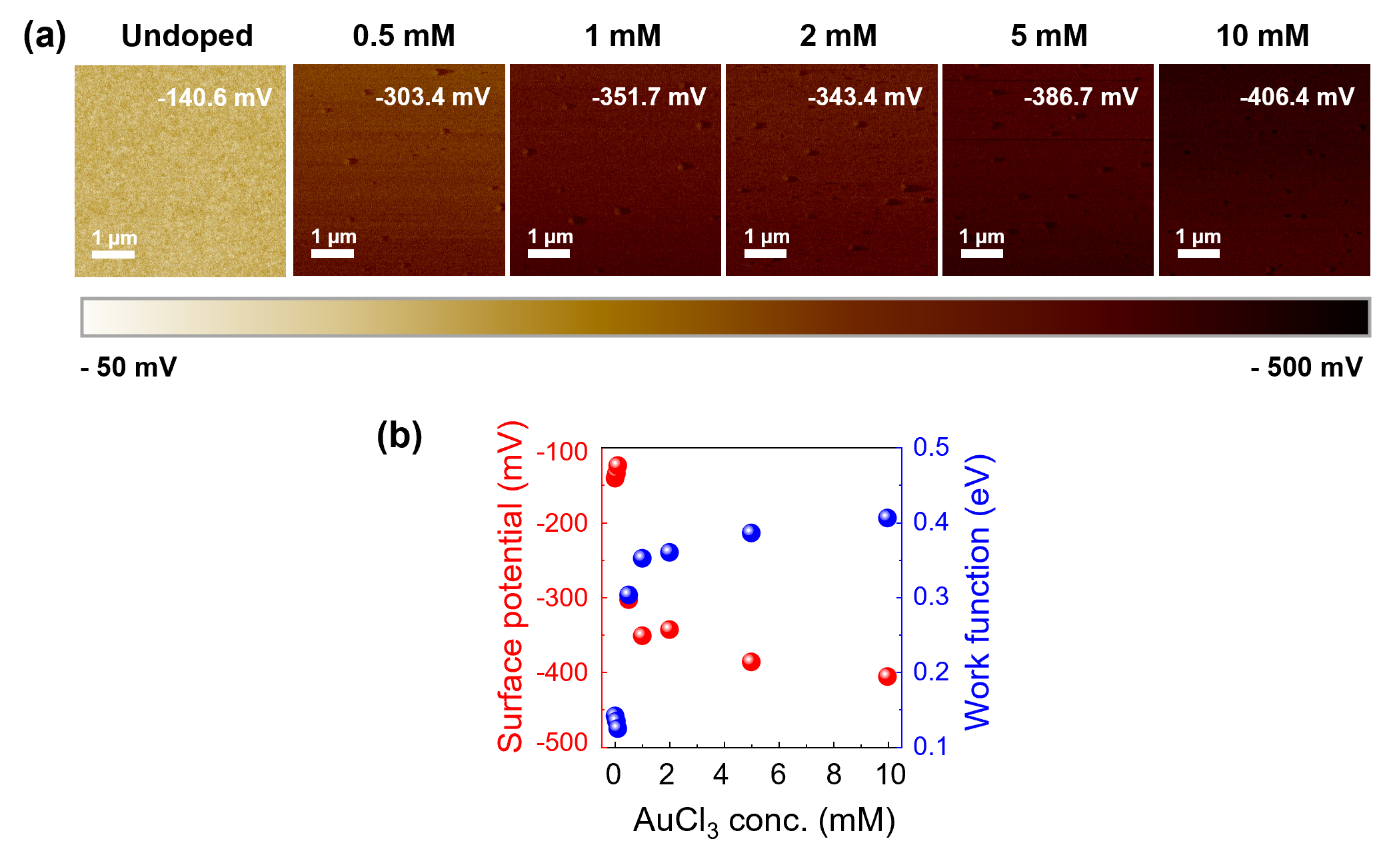


**Figure S10.** (a) KPFM surface profile images, (b) surface potential and work function data of undoped and doped IDTBT films with controlled AuCl_3_ dopant concentration.


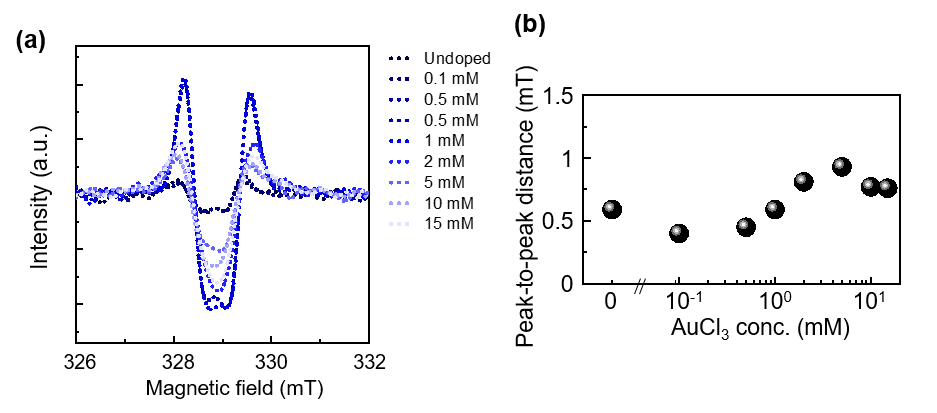


**Figure S11**. (a) ESR 2^nd^ derivative spectra. (b) Calculated peak-to-peak distance of undoped and doped IDTBT films with controlled AuCl_3_ dopant concentration.


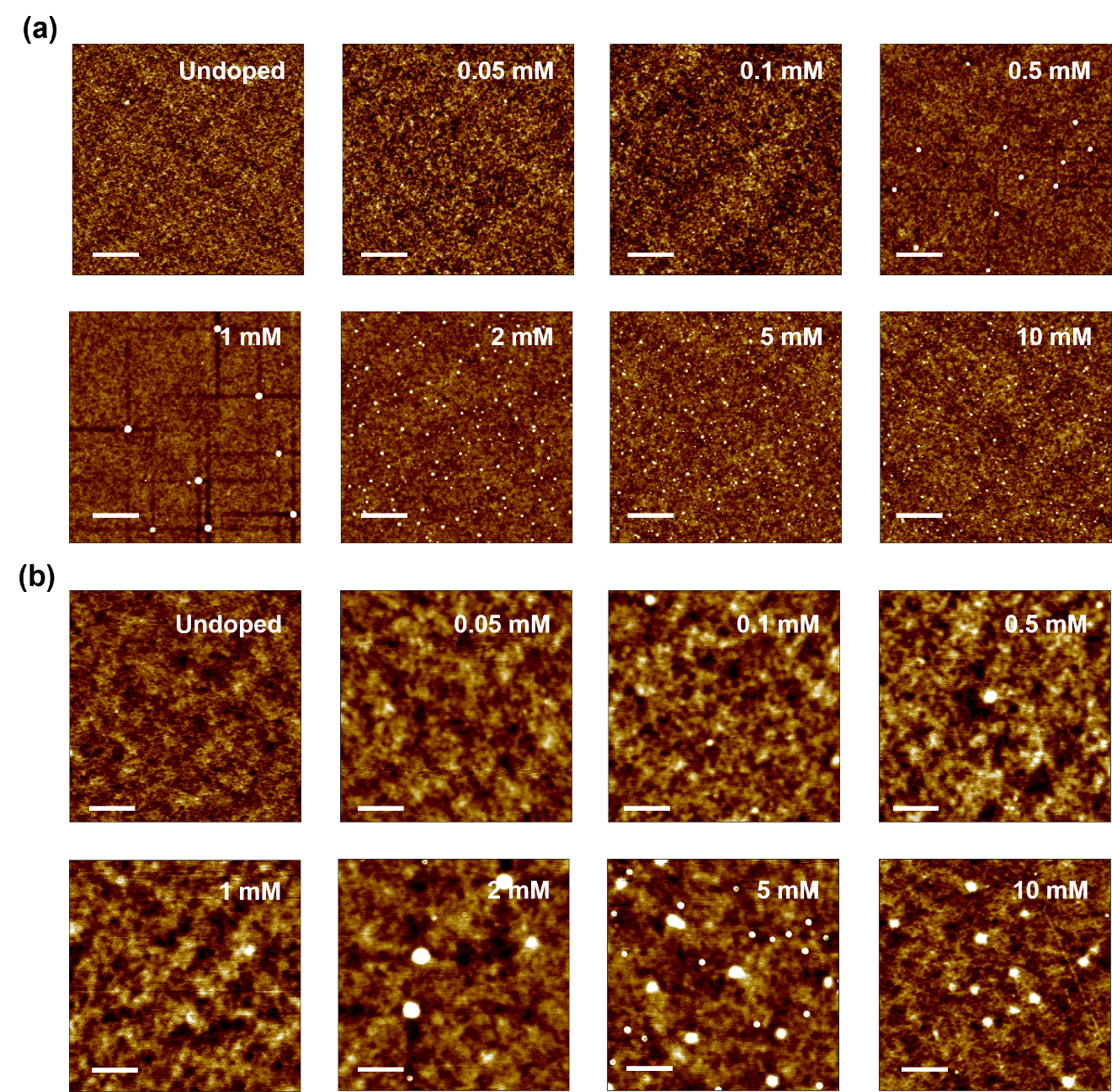


**Figure S12**. AFM height images of undoped and doped IDTBT films with controlled AuCl_3_ dopant concentration. Scale bar represents (a) ~ 1 µm, (b) ~ 200 nm.


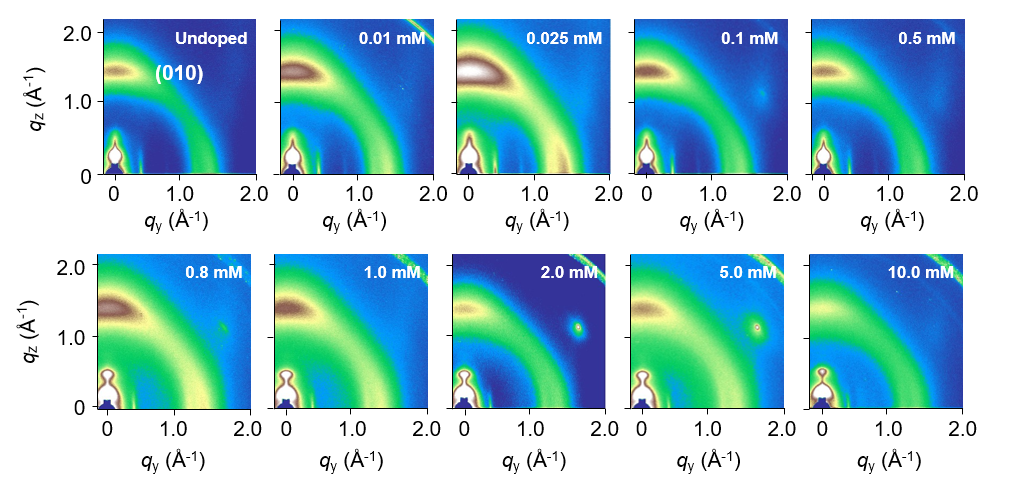


**Figure S13**. 2D GI-WAXS patterns of undoped and doped IDTBT films with controlled AuCl_3_ dopant concentration.


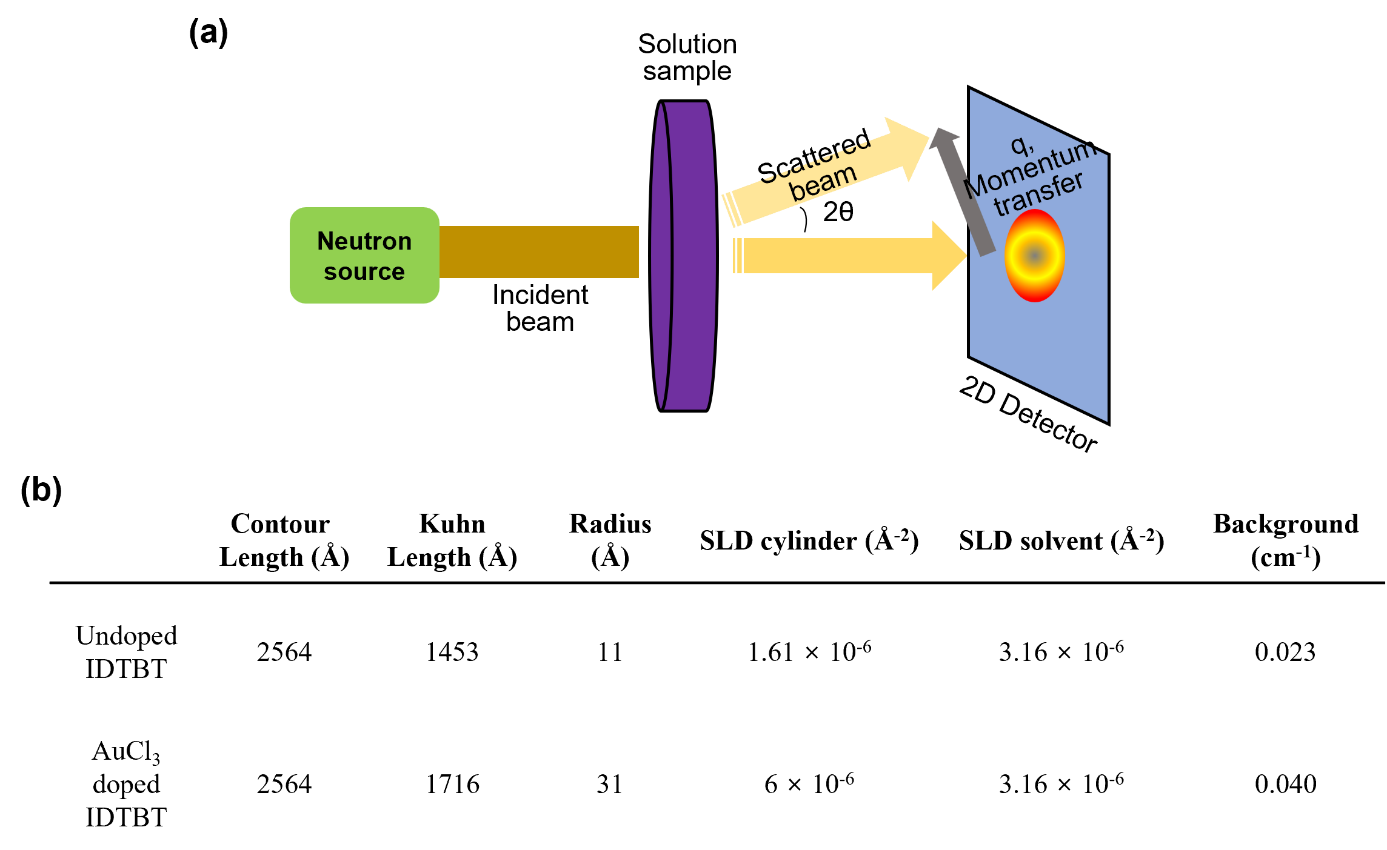


**Figure S14**. (a) Solution SANS experiment geometry. (b) Solution SANS evaluation metrics of pristine IDTBT solution and AuCl_3_ doped IDTBT solution. All plots were fitted by using the SasView cylinder model.


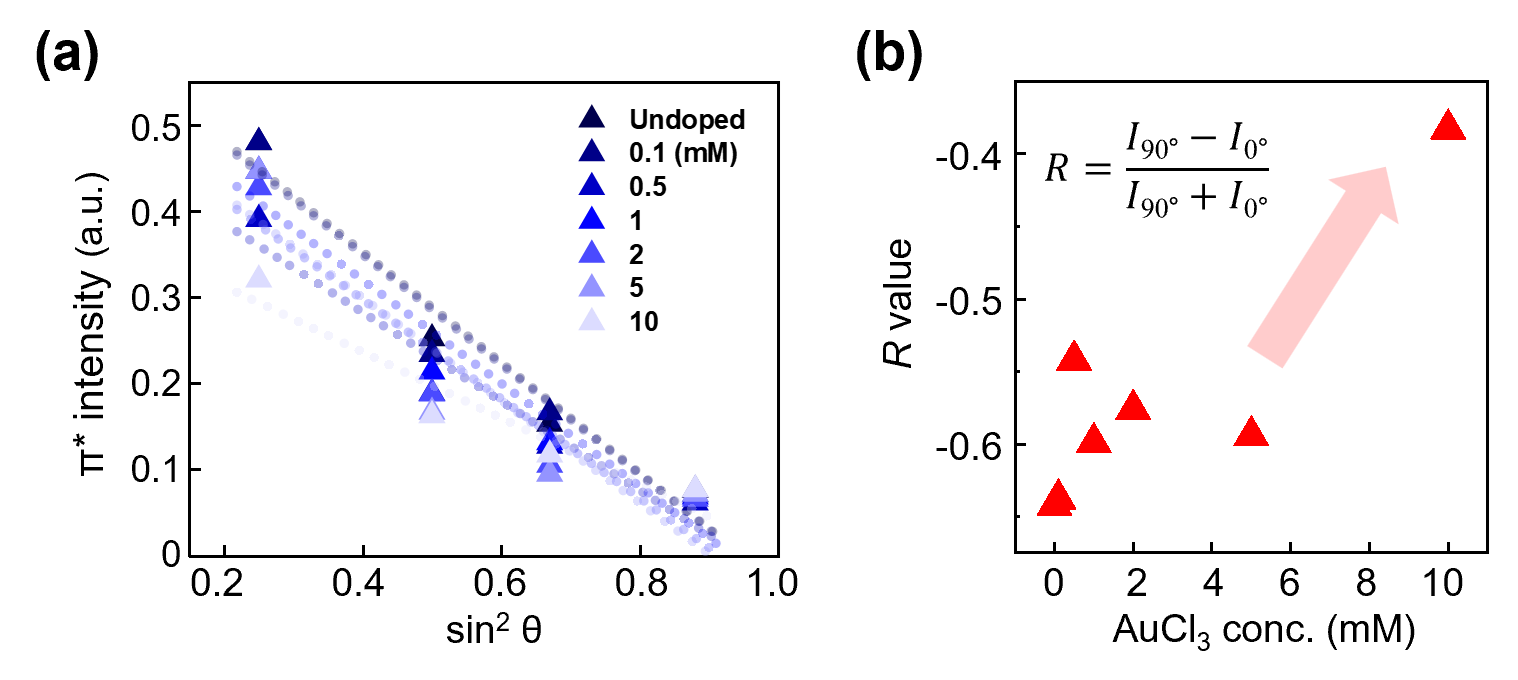


**Figure S15.** (a) Angle-dependent NEXAFS results and (b) *R* values of undoped and doped IDTBT films with controlled AuCl_3_ dopant concentration. *R* values are calculated by each linear slope of the graph (a). The dichroic ratio (*R*) is calculated based on the intensities of the carbon-carbon (C=C) 1s → *π** transition at 285.3 eV, measured at different X-ray incidence angles. *R* is defined as the ratio of intensities at specific angles, where 𝐼_90°_​ and 𝐼_0°_ ​ represent the intensities at 90^°^ and 0^°^ incidence angles, respectively.


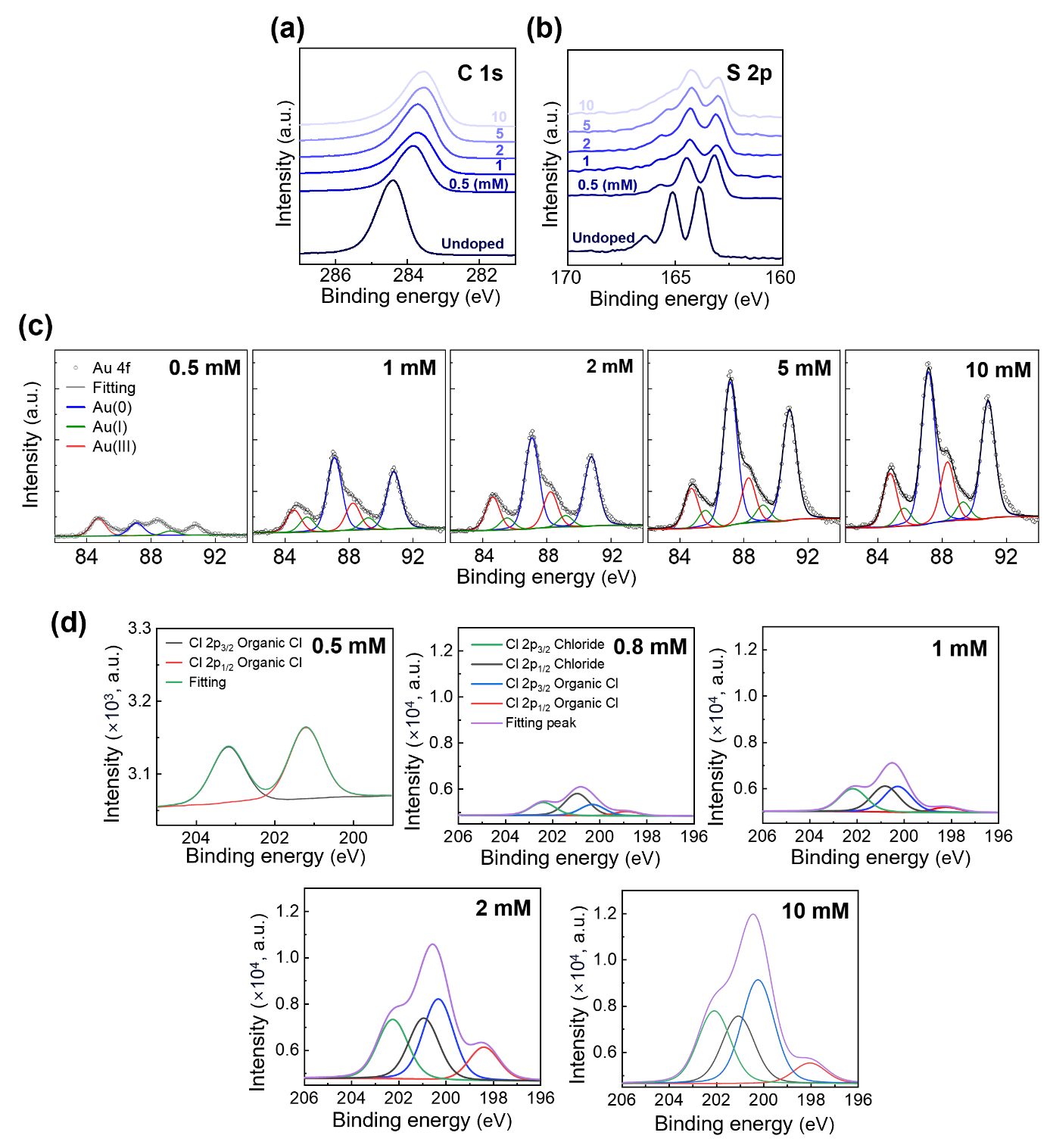


**Figure S16**. (a) C 1s and (b) S 2p XPS spectra of undoped and AuCl_3_-doped IDTBT films. (c) Au 4f XPS spectra with deconvoluted peaks for Au^0^ 4f_5/2_ (red, left)_,_ Au^+^ 4f_5/2_ (green, left)_,_ Au^3+^ 4f_5/2_ (blue, left)_,_ Au^0^ 4f_7/2_ (red, right)_,_ Au^+^ 4f_7/2_ (green, right)_,_ Au^3+^ 4f_7/2_ (blue, right) and fitting peak (gray) of IDTBT films with controlled AuCl_3_ dopant concentration. (d) Cl 2p XPS spectra with deconvoluted peaks for Cl 2p_3/2_ and Cl 2p_1/2_ peaks. Two peaks in lower binding energy (198 eV, 201 eV) correspond to Chloride, and the other two peaks (202 eV, 203 eV) correspond to Organic Cl (C-Cl).

**
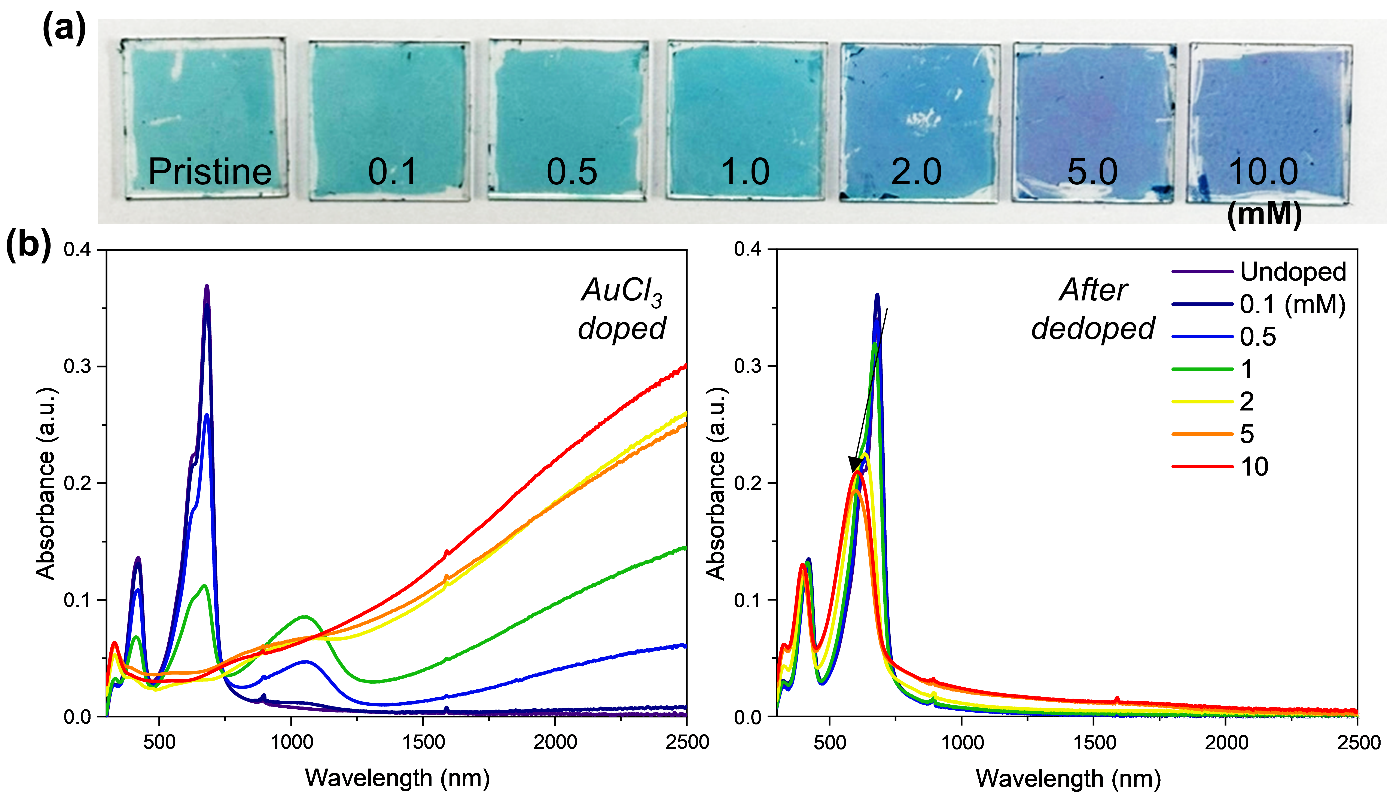
**

**Figure S17**. (a) Photographs of AuCl_3_-doped IDTBT thin-film dedoping processes with dry thermal annealing under harsh condition (180 °C and ~10^-4^ Torr). (b) UV-vis-NIR spectra for doped- (left) and after dedoped- (right) IDTBT thin films.

- XPS after AuCl_3_ 10 mM doping


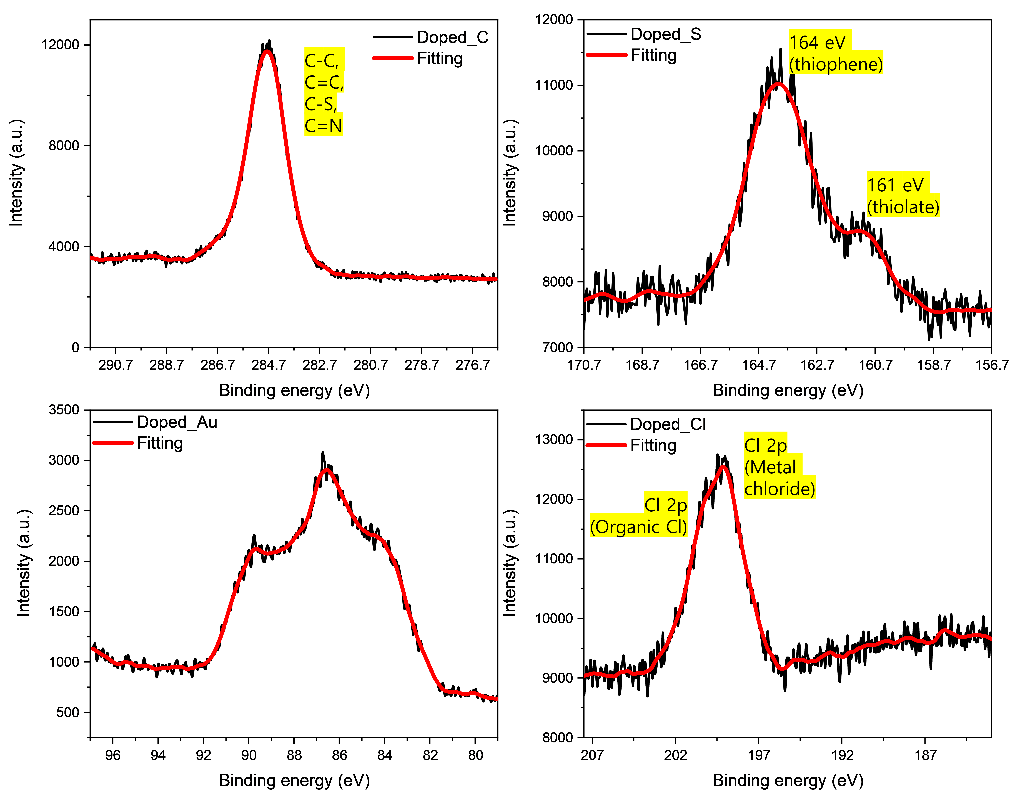


- XPS after dedoping under harsh condition


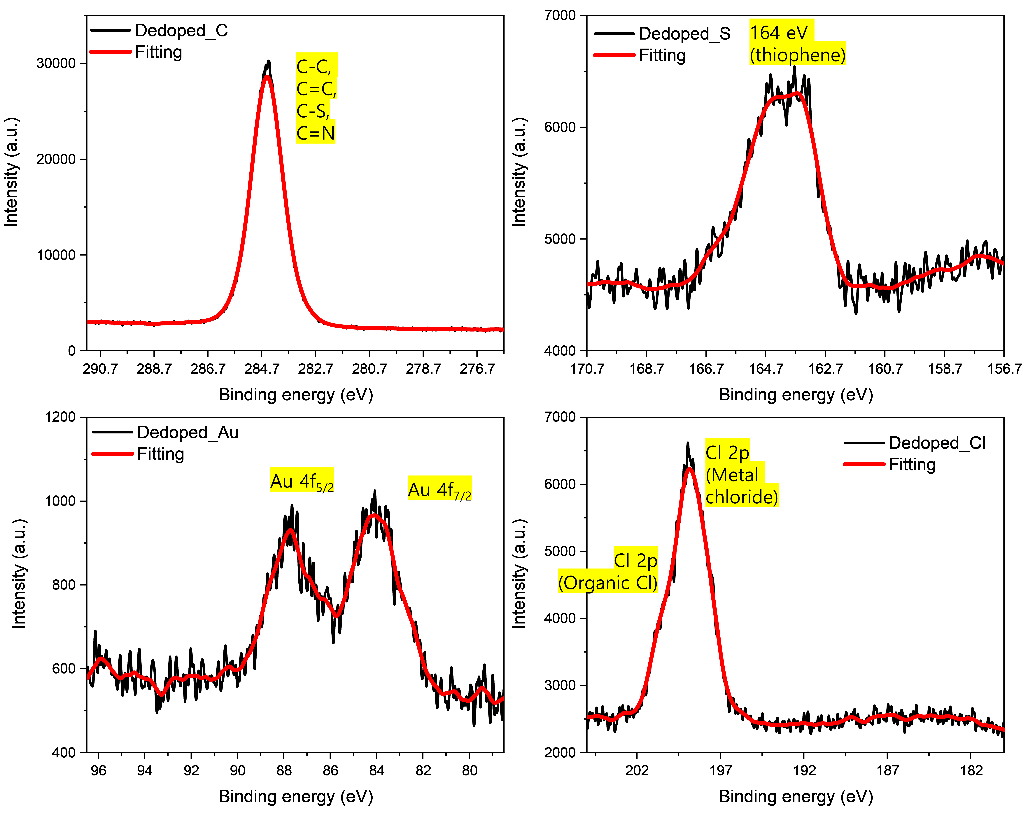


**Figure S18**. XPS spectra of AuCl_3_ doped- (4 graphs (C, S, Cl, and Au) on left side) and dedoped- (4 graphs (C, S, Cl, and Au) on right side) IDTBT thin films.


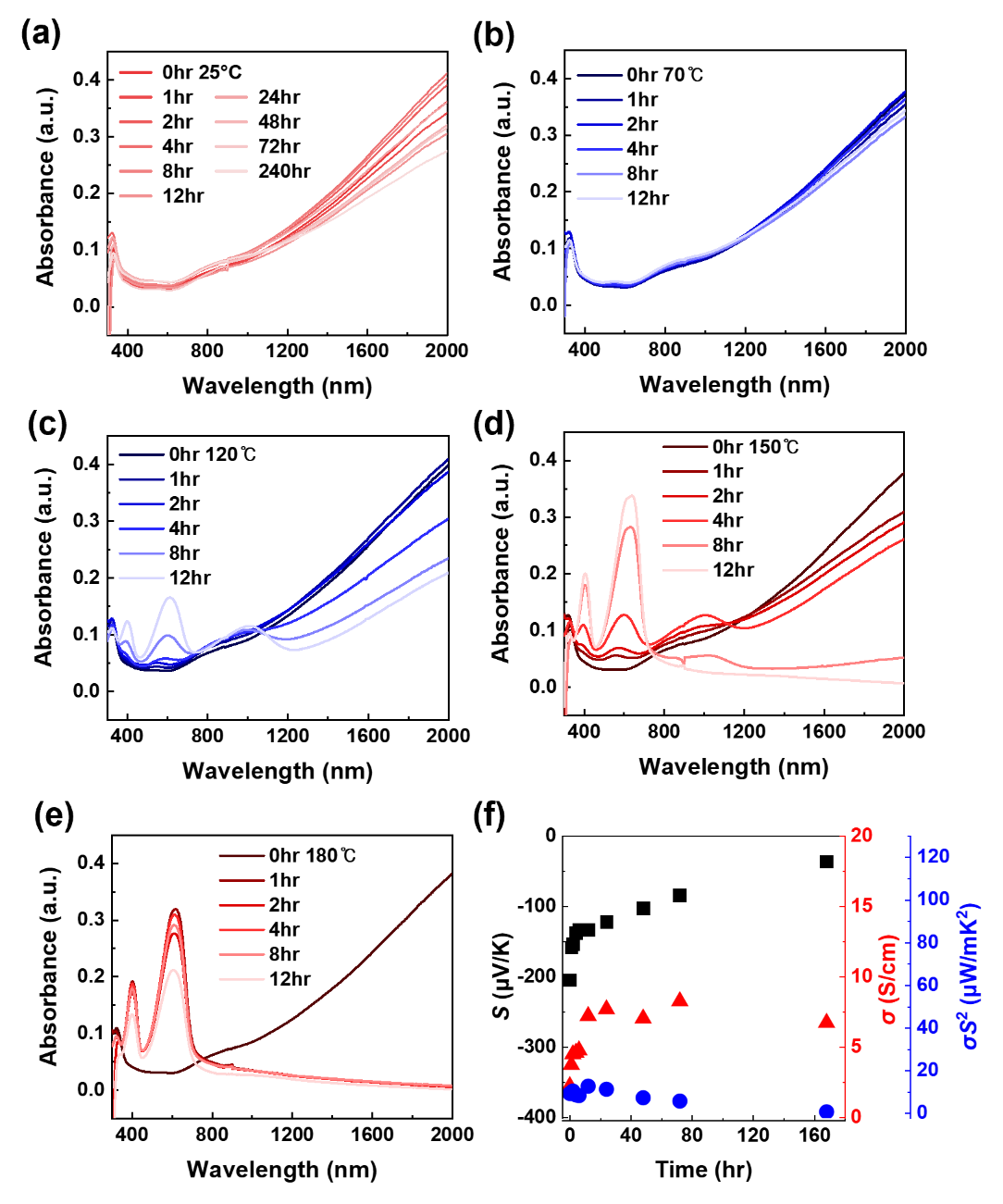


**Figure S19.** Absorbance changes of UV-vis-NIR spectrum of AuCl_3_ 5 mM doped IDTBT films over time at (a) 25 ℃ (b) 70 ℃ (c) 120 ℃ (d) 150 ℃ (e) 180 ℃. At each different temperature, post-doping annealing treatment for dedoping was conducted. Dedoping was progressed until 12hr (240 hr at 25 ℃), to confirm annealing temperature deterioration for 1st device layer. (f) Seebeck coefficient (*S*), electrical conductivity (*σ*), and power factor (*σS*^2^) trends of AuCl_3_ 5 mM doped IDTBT films over time at 25 ℃.


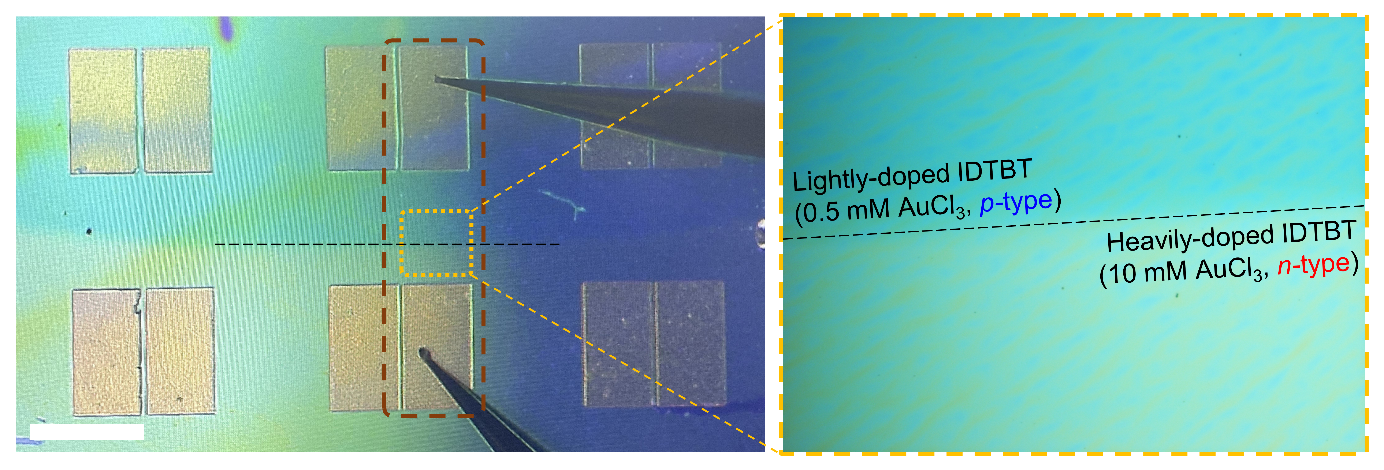


**Figure S20**. Lateral organic homojunction *p-n* diode fabricated from gradient-doped IDTBT. (Scale bar ~ 1 mm)


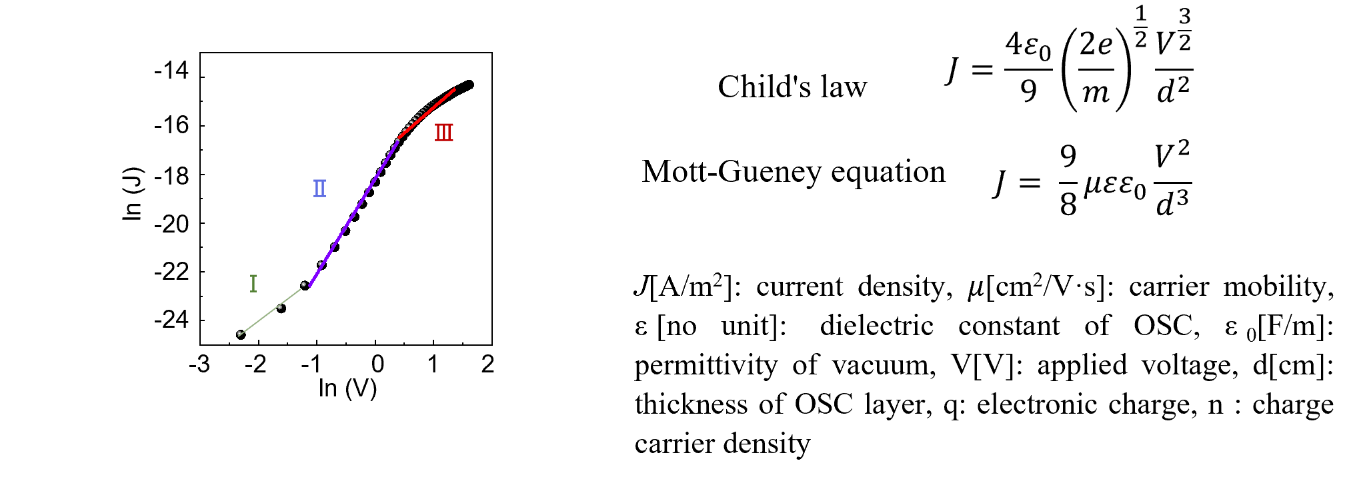


**Figure S21**. ln(*V*)-ln(*J*) plot of *p*-*n* diode data.

**Note:** This shows the linear fitting of the ln(*I*)-ln(*V*) curve, distinguishing three different slope regions. In the region (region Ⅰ), the slope is less than 2, and the current-voltage characteristics adhere to Child’s law. As the applied voltage rises, the dominance shifts towards the injected carriers, leading to the observation of a current limited by trap filling. In the trap-filling step (region Ⅱ), the slope is much larger than 2. Here, within the Trap-limited Space Charge Limited Current (SCLC) region, externally injected charge carriers surpass those internally generated. Current varies quadratically with voltage due to the space-charge-limited effect. Additionally, the presence of traps near the *E*_F_ influences carrier behavior. In the Trap-free SCLC region (region Ⅲ), the slope is 2, and externally injected charge carriers predominate. The current value follows the Mott-Gurney law, displaying a quadratic relationship with voltage. Traps near the *E*_F_ become saturated with charge carriers, diminishing their influence on carrier behavior.


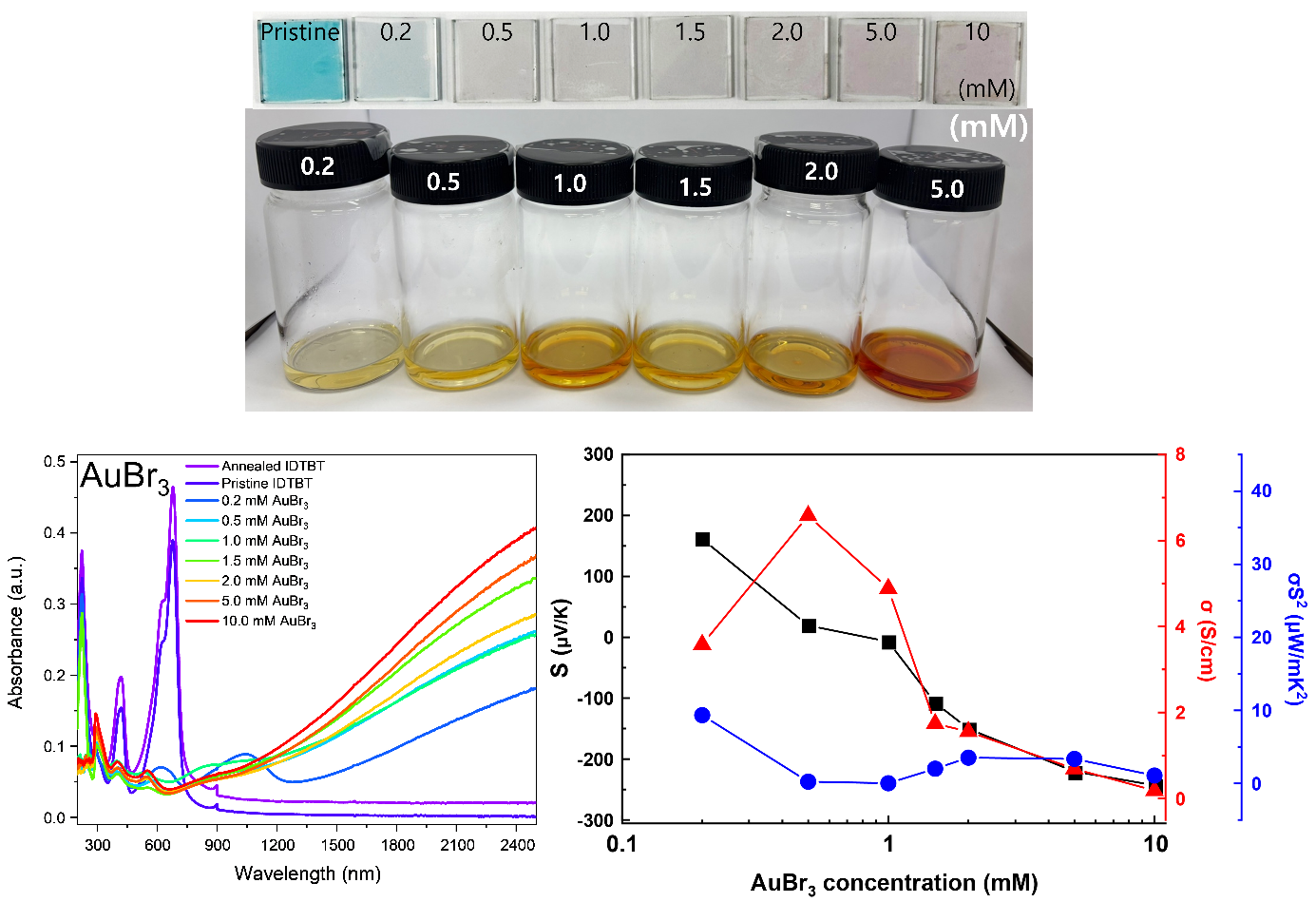


**Figure S22**. Preparation of AuBr_3_ solutions in ACN at various concentrations (mM) and sequential doping of IDTBT thin films, showing the color changes of both the IDTBT films and AuBr_3_ solutions after doping. **(top)** UV-vis-NIR spectra of IDTBT thin films doped with AuBr_3_ and their thermoelectric properties of the doped films, including the *p-n* polarity switching.


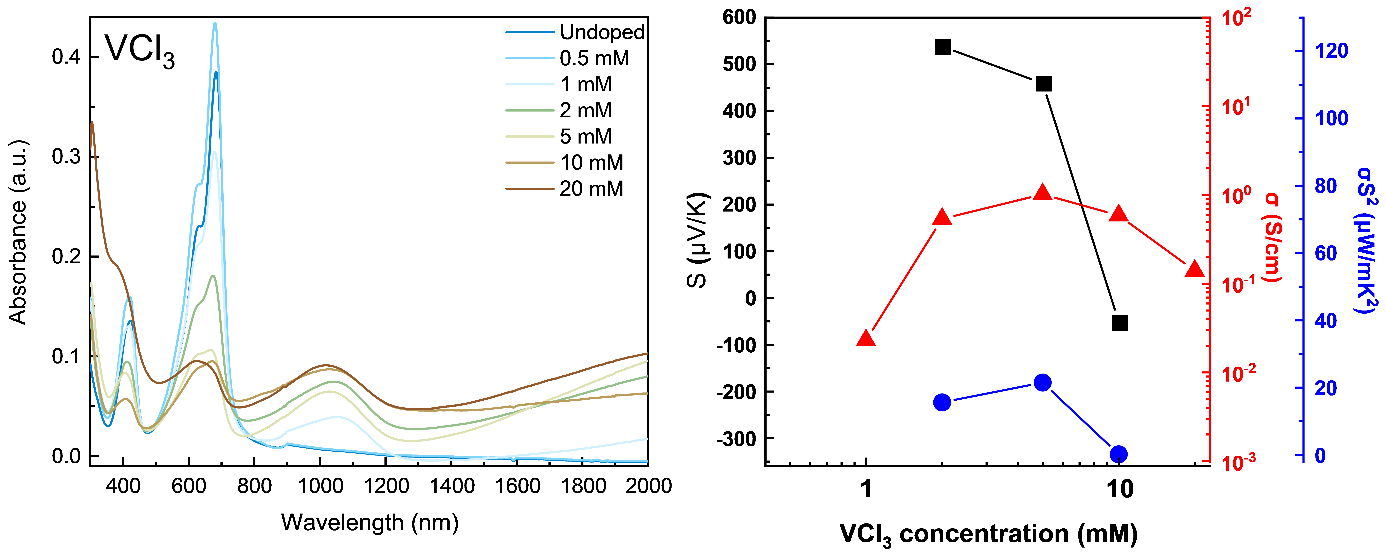


**Figure S23**. UV-vis-NIR spectra of IDTBT thin films sequentially doped with VCl_3_ and their thermoelectric properties of the doped films, including the *p-n* polarity switching.


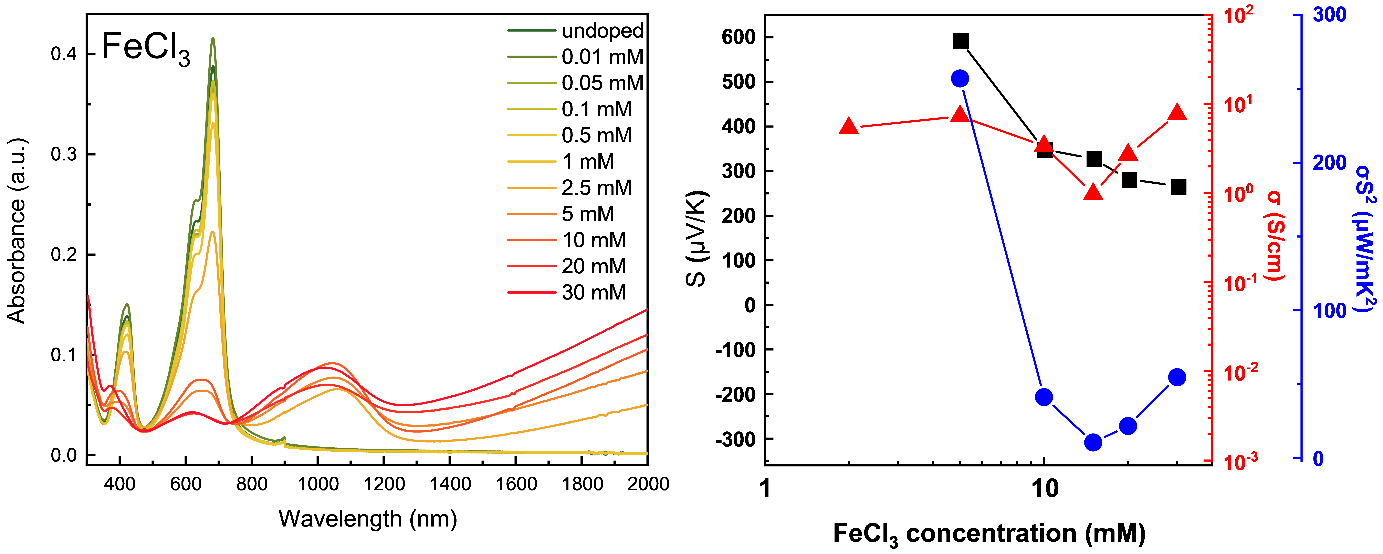


**Figure S24**. UV-vis-NIR spectra of IDTBT thin films sequentially doped with FeCl_3_ and their thermoelectric properties of the doped films, including the *p-n* polarity switching.


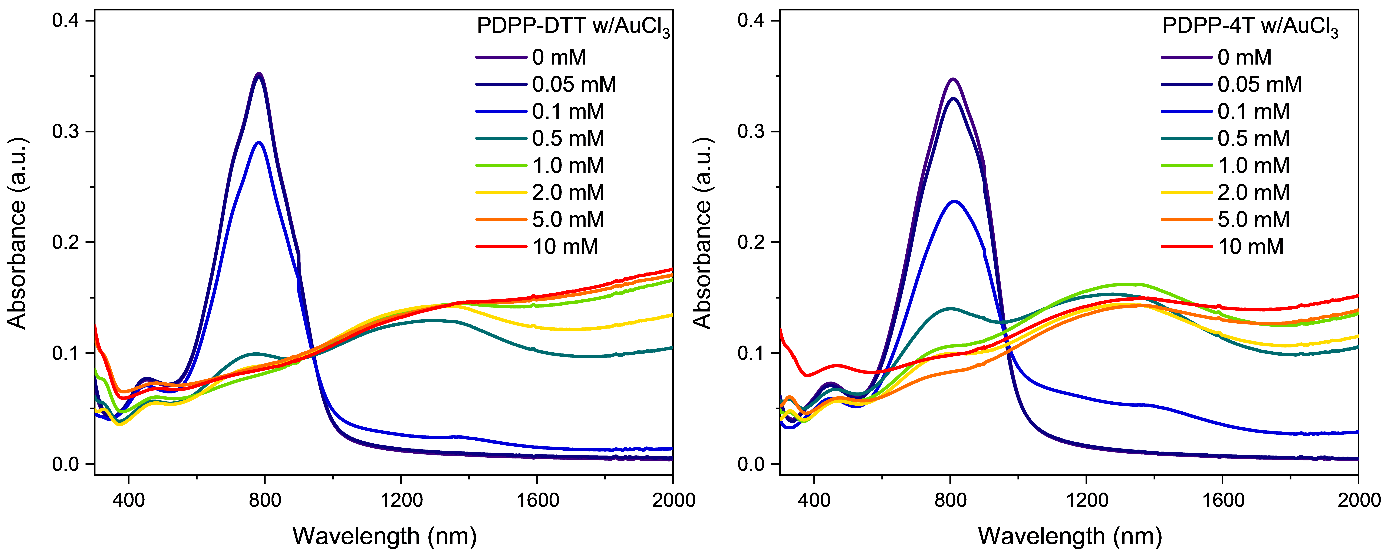


**Figure S25**. UV-vis-NIR spectra of PDPP-DTT and -4T thin films sequentially doped with AuCl_3_.

**Supplementary Notes**

**Note S1.** Electrochemical redox chemistry under AuCl_3_ doping process.

Au_2_Cl_6_ (*s*) + ACN (*l*) → 2AuCl_3_ (ACN) − (1)

Cation: [AuCl_2_(ACN)_2_]^+^ or [AuCl(ACN)_3_]^2+^− (2-1)

Anion: Cl^-^ (major counter ion) or [AuCl_4_]^-^ (under high conc. of Cl^-^)− (2-2)

[AuCl_4_]^⁻^ + 3e^⁻^ → Au (*s*) + 4Cl^⁻^ *E*° = +1.002 V (vs. SHE) − (3)

[AuCl_2_]^⁻^ + e^⁻^ → Au (*s*) + 2Cl^⁻^ *E*° = +1.154 V (vs. SHE) − (4)

AuCl_3_ dopant exists as a dimer (Au_2_Cl_6_) in the solid state. Upon dissolution, AuCl_3_ is known to dissociate into monomeric species through strong Lewis’s acid-base interactions, particularly in the presence of high dielectric constant solvents such as acetonitrile (ACN, ε = 37.5). ACN not only facilitates the dissolution of ionic species but also stabilizes gold(III) complexes, which accounts for the high solubility of AuCl_3_ (125 g/L at r.t.) and its widespread use in doping studies. In solution, AuCl_3_ can form complexes with ACN in a two-step mechanism: (1) Initially, the cyano group (–C≡N) of ACN coordinates to the gold(III) center, forming an intermediate [AuCl_3_ (ACN)]. This process involves the sharing of electron pairs with the vacant d-orbitals of gold(III), leading to the formation of stable complexes. During this process, some chloride ions (Cl⁻) may dissociate, resulting in the prevalence of cationic species such as [AuCl_2_ (ACN)_2_]⁺ or [AuCl(ACN)_3_]^2+^. To further support the thermodynamic feasibility of electron transfer, we compared the HOMO level of IDTBT (–5.22 eV, as measured in our study) to the standard hydrogen electrode (SHE) scale, yielding *E*_HOMO_ = (–5.22 + 4.44) V = –0.78 V (vs. SHE). Comparing this to the reduction potential of AuCl_4_^⁻^ (*E*° = +1.002 V vs. SHE), the potential difference is *ΔE* = *E*°_AuCl4⁻_ – *E*_HOMO_ = 1.002 – (–0.78) = 1.782 V, indicating a highly favorable thermodynamic driving force for electron transfer from IDTBT to AuCl_4_^⁻^ (*ΔG* = –*nFE*°). This supports the notion that AuCl_4_^⁻^ can readily oxidize IDTBT, enabling efficient charge-transfer doping.

**Note S2.** Universality of polarity switching through heavy AuCl_3_ doping.

The universality of our method is rooted in the alignment between the polymer’s LUMO level and the dopant’s redox potential. Specifically, *p-n* polarity switching occurs when the dopant’s reduction potential (vs. SHE) is higher than or comparable to the polymer’s LUMO-derived potential (−3.52 eV, +0.92 V vs. SHE). To validate this, we tested alternative Lewis’s acids: VCl_3_ (+0.80 V vs. SHE) and AuBr_3_ exhibited *p-n* polarity switching akin to AuCl_3_, driven by balanced hole/electron generation and mobile Cl⁻/Br⁻ ions (**Figures S22** and **S23**). However, FeCl_3_ (+0.77 V vs. SHE) failed to induce this transition, likely due to its weaker oxidizing power (limiting electron injection) and stronger Fe-Cl bonds, which hinder ion mobility and stabilize *p*-doping (**Figure S24**). Notably, while VCl_3_ and AuBr_3_ replicated the *p-n* polarity switching, their power factors (PFs) were lower than AuCl_3_-doped IDTBT.

We attribute this to gold’s ultra-low ionization tendency, which enhances charge-carrier injection efficiency at the metal-polymer interface. Regarding polymer substitution, while IDTBT was our model system, the methodology should generalize to other polymers with LUMO levels ≤ −3.52 eV. For instance, polymers like DPP-based or NDI-based derivatives could be candidates if their electronic structures align. However, such substitutions were beyond this study’s scope, as our focus was establishing the AuCl_3_-IDTBT system as a foundational case. Future work will explore dopant-polymer pairings to optimize both electronic and ionic contributions.

To the best of our knowledge, while there are numerous reports in the literature on doping conjugated polymers with AuCl_3_, IDTBT remains the only system in which a *p-n* polarity switching has been mechanistically elucidated. No other studies to date have provided a detailed mechanistic investigation of the *p-n* polarity switching induced by AuCl_3_ doping in conjugated polymers.^[^**^S2^**^-^**^S7^**^]^

As part of our extended investigation, we additionally tested AuCl_3_ doping on PDPP-DTT and PDPP-4T conjugated polymers with AuCl_3_ (**Figure S25**). However, comparative analysis of NIR absorbance spectra revealed less pronounced polaron/bipolaron formation in these systems compared to IDTBT, as evidenced by weaker absorption features in the NIR region. While this observation suggests intrinsic differences in doping efficiency or charge-carrier stabilization mechanisms among these polymers, the exact origin of this disparity—whether due to variations in backbone rigidity, side-chain effects, or LUMO level alignment—remains unresolved. We acknowledge this as an important open question and plan to systematically explore these structure-property relationships in future studies.

**Supplementary Tables**

**Table S1**. Reported other studies in which regard the Seebeck coefficient inversion or polarity switching.

| **Type** | **Polymer** | **Dopant** | **Doping method** | **Switching Evidence** | **Mechanism** | | **Structural Insight** | **Ref.** |
| --- | --- | --- | --- | --- | --- | --- | --- | --- |
| *p*🡪*n* | PDPP-4T,  PDPP-T-TT-T,  PBTII-T2,  PbisBTII-T2F2,  PCDTBT | FeCl_3_, NOBF_4_ | Solution mixed doping | Seebeck sign inversion. AC Hall | Mixed-phase transport: delocalized / hopping | |  | [S8] |
| *p*🡪*n* | DPP-TTT | FeCl_3_ | Solution mixed doping | Seebeck sign inversion | Fermi level drop via deep oxidation | XPS | | [S9] |
| *p*🡪*n* | PAQM2T-TVT,  PDPP2T-TVT | FeCl_3_,  Magic Blue | Sequential doping | Seebeck sign inversion | Crystal doping essential for switching | TEM | | [S10] |
| *p*🡪*n* | PDPP-5T | FeCl_3_ | Sequential doping | Seebeck sign inversion | Band filling, DOS curvature |  | | [S11] |
| *n*🡪*p* | BBL | PSSNa | Electrochemical doping | Seebeck sign inversion | Coulomb gap, multi-state formation | Raman | | [S12] |
| *n*🡪*p* | PNDI2TEG-2T | N-DMBI | Solution mixed doping | Seebeck sign inversion | CTC transport from LUMO filling |  | | [S13] |
| *n*🡪*p* | P(PymPh) | NaNap | Sequential doping | Seebeck sign inversion | Fermi level shift via DOS shaping | XPS | | [S14] |
| *p*🡪*n* | PPDT2FBT-based  polymers | [PMIM]^+^ [TFSI]^−^ | Electrochemical doping | Seebeck sign inversion | DOS modulation in electrochemical setup | GIWAXS | | [S15] |
| *p*🡪*n* | IDTBT | AuCl₃ | Sequential doping | Seebeck sign inversion, AC Hall, FET | Covalent chlorination, band structure change | GIWAXS, XPS, Raman, NEXAFS | | This work |

**Table S2**. Reported other studies in which regard the chlorination usages from metal halides dopants for various nano- and conjugated materials.

| **Materials** | **Dopants** | **Chlorination usages** | **References** |
| --- | --- | --- | --- |
| SWCNT | AuCl_3_ | Chemical doping | [S16] |
| CNT | AuCl_3_ | Chemical doping | [S17] |
| P[(DHP-EDOT)-bis-EDOT) | FeCl_3_ | Chemical doping | [S18] |
| TDPP | FeCl_3_ | oCVD synthesis process | [S19] |
| PEDOT | FeCl_3_ | Chemical vapor doping | [S20] |
| PEDOT | FeCl_3_, SbCl_5_, VOCl_3_ | Chemical vapor doping | [S21] |
| P3AT  (PHTT, PDDTT) | FeCl_3_ | Oxidative polymerization | [S22] |
| PVDF | FeCl_3_ | Chemical doping | [S23] |
| Polyacethylene | FeCl_3_ | Chemical doping | [S24] |
| polyphenylacetylene | FeCl_3_ | Chemical doping | [S25] |

**Table S3**. Concentration unit conversion table from mM to wt. % unit for sequential doping IDTBT thin-film dipping with AuCl_3_ ‘X’ g in 5 mL ACN (*d* = 0.786 g/L).

| ***AuCl_3_ conc.***  ***(mM)*** | *AuCl_3_ weight, X*  *(g)* | *Total weight*  *(g)* | ***AuCl_3_ conc.***  ***(wt. %)*** |
| --- | --- | --- | --- |
| ***0.01*** | *0.00015* | *3.93015* | ***0.00387*** |
| ***0.05*** | *0.00076* | *3.93076* | ***0.01928*** |
| ***0.10*** | *0.00152* | *3.93152* | ***0.03858*** |
| ***0.50*** | *0.00758* | *3.93758* | ***0.1926*** |
| ***0.80*** | *0.01213* | *3.94213* | ***0.3077*** |
| ***1.00*** | *0.01517* | *3.94517* | ***0.3845*** |
| ***2.00*** | *0.03033* | *3.96033* | ***0.7657*** |
| ***5.00*** | *0.07583* | *4.00583* | ***1.8925*** |
| ***10.00*** | *0.15166* | *4.08166* | ***3.7151*** |
| ***15.00*** | *0.22749* | *4.15749* | ***5.4723*** |

**Table S4**. AC Hall measurement parameters doped IDTBT films with controlled AuCl_3_ dopant concentration.

|  | **0.8 mM** | **1.0 mM** | **2.0 mM** | **10.0 mM** |
| --- | --- | --- | --- | --- |
| Carrier type | *p* | *p* | *n* | *n* |
| Hall voltage [V] | 6.04 × 10^-7^ | 3.10 × 10^-7^ | **-**1.61 × 10^-7^ | **-**1.47 × 10^-6^ |
| Hall coefficient [cm³/C] | 2.84 × 10^-2^ | 1.75 × 10^-2^ | 7.34 × 10^-3^ | 6.98 × 10^-3^ |
| Resistivity [Ω∙cm] | 4.44 × 10^-1^ | 1.98 × 10^-1^ | 3.59 × 10^-1^ | 3.35 × 10^-1^ |
| Hall mobility [cm²/V∙s] | 6.38 × 10^-2^ | 8.87 × 10^-2^ | 2.04 × 10^-2^ | 2.09 × 10^-2^ |
| Carrier concentration [1/cm³] | 2.20 × 10^20^ | 3.56 × 10^20^ | 8.51 × 10^20^ | 8.94 × 10^20^ |

* Each value is averaged with 5 thermoelectric devices.

**Table S5**. GI-WAXS diffraction *q* values and calculated *d*-spacing data in (a) in-plane and (b) out-of-plane direction peaks of undoped and doped IDTBT films with controlled AuCl_3_ dopant concentration.

(a) In-plane

| Doping concentration (mM) | | *q*_001_  (nm^-1^) | *d*-spacing  (nm) | *q*_002_  (nm^-1^) | *d*-spacing  (nm) |
| --- | --- | --- | --- | --- | --- |
| Undoped | 3.9 | | 1.61 | 7.9 | 1.59 |
| 0.01 | 4.0 | | 1.57 | 7.9 | 1.59 |
| 0.025 | 4.0 | | 1.57 | 7.9 | 1.59 |
| 0.1 | 4.0 | | 1.57 | 7.9 | 1.59 |
| 0.5 | 4.0 | | 1.57 | 7.9 | 1.59 |
| 0.8 | 4.0 | | 1.57 | - | - |
| 1 | 4.0 | | 1.57 | - | - |
| 2 | 4.0 | | 1.57 | - | - |
| 5 | 4.0 | | 1.57 | - | - |
| 10 | 4.0 | | 1.57 | - | - |
| 15 | 4.0 | | 1.57 | - | - |
| 20 | 4.0 | | 1.57 | - | - |

(b) Out-of-plane

| Doping concentration (mM) | *q*_100_  (nm^-1^) | *d*-spacing  (nm) | *q*_200_  (nm^-1^) | *d*-spacing  (nm) | *q*_010_  (nm^-1^) | *π-π* stacking  (nm) |
| --- | --- | --- | --- | --- | --- | --- |
| Undoped | 3.3 | 1.90 | - | - | 15.4 | 0.41 |
| 0.01 | 3.3 | 1.90 | - | - | 15.4 | 0.41 |
| 0.025 | 3.3 | 1.90 | - | - | 15.3 | 0.41 |
| 0.1 | 3.3 | 1.90 | - | - | 15.2 | 0.41 |
| 0.5 | 3.3 | 1.90 | - | - | 15.2 | 0.41 |
| 0.8 | 3.0 | 2.09 | 5.7 | 2.20 | 15.1 | 0.42 |
| 1 | 2.9 | 2.17 | 5.6 | 2.24 | 15.1 | 0.42 |
| 2 | 2.8 | 2.24 | 5.5 | 2.28 | 15.0 | 0.42 |
| 5 | 2.8 | 2.24 | 5.4 | 2.33 | 14.9 | 0.42 |
| 10 | 2.8 | 2.24 | 5.4 | 2.33 | 15.0 | 0.42 |
| 15 | 2.8 | 2.24 | 5.5 | 2.28 | 15.1 | 0.42 |
| 20 | 2.8 | 2.24 | 5.3 | 2.37 | 15.0 | 0.42 |

**Table S6.**A comparison of *RR* values with reported organic homojunction diodes using conjugated polymers.

| **Year** | **Material** | **Structure** | ***RR*** | **Ref.** |
| --- | --- | --- | --- | --- |
| 2022 | PEDOT:PSS | Vertical | 3 | [S26] |
| 2021 | FeCl_3_ doped  T2-DPPT, DPPTTT, T-DPPT | Lateral | 2100 | [S27] |
| 2014 | PPV | Lateral | 120 | [S28] |
| 2025 | AuCl_3_ doped  IDTBT | Vertical | 58270 | This work |

**Supplementary Reference**

[S1] A. Salleo, T. D. Anthopoulos, H. Sirringhaus, I. McCulloch, *J. Am. Chem. Soc.* **2020**, *142*, 652.
[S2] M. S.A. Abdou, S. Holdcroft, *Synth. Met.* **1993**, 60, 93.

[S3] M. S. A. Abdou, S. Holdcroft, *Chem. Mater.* **1996**, 8, 26.

[S4] Y. H. Kang, S.-J. Ko, M.-H. Lee, Y. K. Lee, B. J. Kim, S. Y. Cho, *Nano Energy* **2021**, 82, 105681.

[S5] S. E. Yoon, J. Park, J. E. Kwon, S. Y. Lee, J. M. Han, C. Y. Go, S. Choi, K. C. Kim, H. Seo, J. H. Kim, B.-G. Kim, *Adv. Mater.* **2020**, 32, 2005129.

[S6] D. Y. Lee, D. E. Choi, Y. Ahn, H. Kye, M. S. Kim, B.-G. Kim, *Polymers* **2024**, 16, 1884.

[S7] H. Lee, H. Kim, H. Jin, S. Kang, T. W. Yoon, D. Lee, G. Zhang, M. Kim, B. Kang, *Adv. Sci.* **2025**, 12, 2412663.
[S8] Z. Liang, H. H. Choi, X. Luo, T. Liu, A. Abtahi, U. S. Ramasamy, J. A. Hitron, K. N. Baustert, J. L. Hempel, A. M. Boehm, A. Ansary, D. R. Strachan, J. Mei, C. Risko, V. Podzorov, K. R. Graham, *Nat. Mater.* **2021**, *20*, 518.
[S9] J. Wang, Y. Wang, Q. Li, Z. Li, K. Li, H. Wang, *CCS Chem.* **2021**, *3*, 2482.
[S10] B. Dyaga, A. Lemaire, S. Guchait, H. Zeng, B. Schmaltz, M. Brinkmann, *J. Mater. Chem. C* **2023**, *11*, 16554.
[S11] H. Zeng, M. Mohammed, V. Untilova, O. Boyron, N. Berton, P. Limelette, B. Schmaltz, M. Brinkmann, *Adv. Electron. Mater.* **2021**, *7*, 2000880.
[S12] K. Xu, T.-P. Ruoko, M. Shokrani, D. Scheunemann, H. Abdalla, H. Sun, C.-Y. Yang, Y. Puttisong, N. B. Kolhe, J. S. M. Figueroa, J. O. Pedersen, T. Ederth, W. M. Chen, M. Berggren, S. A. Jenekhe, D. Fazzi, M. Kemerink, S. Fabiano, *Adv. Funct. Mater.* **2022**, *32*, 2112276.
[S13] J. Liu, G. Ye, B. v. d. Zee, J. Dong, X. Qiu, Y. Liu, G. Portale, R. C. Chiechi, L. J. A. Koster, *Adv. Mater.* **2018**, *30*, 1804290.
[S14] S. Hwang, W. J. Potscavage, Y. S. Yang, I. S. Park, T. Matsushima, C. Adachi, *Phys. Chem. Chem. Phys.* **2016**, *18*, 29199.
[S15] W. Choi, S. Kim, S. Lee, C. Jung, A. Tripathi, Y. Lee, H. Y. Woo, H. Lee, *Small Methods* 2023, 7, 2201145.
[S16] S.-M. Yoon, U. J. Kim, A. Benayad, I. H. Lee, H. Son, H.-J. Shin, W. M. Choi, Y. H. Lee, Y. W. Jin, E.-H. Lee, S. Y. Lee, J.-Y. Choi, J. M. Kim, ACS Nano **2011**, 5, 1353.

[S17] S. M. Kim, K. K. Kim, Y. W. Jo, M. H. Park, S. J. Chae, D. L. Duong, C. W. Yang, J. Kong, Y. H. Lee, *ACS Nano* **2011**, *5*, 1236.

[S18] S. J. Jeon, N. Zhao, Y. Yuan, Y. Li, *Adv. Funct. Mater.* **2025**, 2424664.

[S19] M. K. Charyton, T. Reiker, K. Kotwica, M. Góra, H. Zacharias, N. D. Boscher, *Mater. Adv.* **2023**, *4*, 2625.

[S20] G. Drewelow, H. Wook Song, Z.-T. Jiang, S. Lee, *Appl. Surf. Sci.* **2020**, *501*, 144105.

[S21] M. Heydari Gharahcheshmeh, M. T. Robinson, E. F. Gleason, K. K. Gleason, *Adv. Funct. Mater.* **2021**, *31*, 2008712.

[S22] J. M. Xu, H. S. O. Chan, S. C. Ng, T. S. Chung, *Synth. Met.* **2002**, *132*, 63.

[S23] A. Tawansi, H. I. Abdel-Kader, W. Balachandran, E. M. Abdel-Razek, *J. Mater. Sci.* **1994**, *29*, 4001.

[S24] K. Akagi, T. Kadokura, H. Shirakawa, *Polymers* **1992**, *33*, 4058.

[S25] M. V. Russo, G. Polzonetti, A. Furlani, *Synth. Met.* **1991**, *39*, 291.

[S26] S. Aboulhadeed, M. Ghali, M. M. Ayad, *Sci. Rep.* **2022**, *12*, 12485.
[S27] J. Wang, Y. Wang, K. Li, X. Dai, L. Zhang, H. Wang, *Adv. Mater.* **2022**, *34*, 2106624.

[S28] J. Liu, I. Engquist, M. Berggren, *ACS Appl. Mater. Interfaces* **2014**, 6, 13266.
